# Frozen Interphase Domain and Mechanism of the Snakelike Macroscopic Motion in a Dynamic Crystal Solvate

**DOI:** 10.1021/jacs.6c02665

**Published:** 2026-04-27

**Authors:** Emmanuele Parisi, Fabio Borbone, Elena Simone, Luca Catalano, Durga Prasad Karothu, Sanjit Manohar Majhi, Ejaz Ahmed, Salvatore Zarrella, Timothy M. Korter, Roberto Centore

**Affiliations:** a Department of Applied Science and Technology, 19032 Politecnico of Turin, I-10129 Turin, Italy; b Department of Chemical Sciences, University of Naples Federico II, Via Cintia, I-80126 Naples, Italy; c Dynamic Molecular Materials Laboratory, Department of Life Sciences, 9306 University of Modena and Reggio Emilia, Via G. Campi 103, 41125 Modena, Italy; d Center for Smart Engineering Materials, 167632 New York University Abu Dhabi, PO Box, 129188, Abu Dhabi, United Arab Emirates; e Smart Materials Lab, 167632 New York University Abu Dhabi, P.O. Box, 129188, Abu Dhabi, United Arab Emirates; f Department of Chemistry, 2029 Syracuse University, 111 College Place, Syracuse, New York 13244-4100, United States

## Abstract

Dynamic molecular crystals capable of structural transformations have significant potential in functional materials. In this study, we investigate the phase transitions and mechanical behavior of 4-hydroxy-*N*′-(4-methylbenzylidene)­benzohydrazide *N*-methylpyrrolidin-2-one solvate (HMBB·NMP). Thermal analysis and single-crystal X-ray diffraction reveal the existence of three solvate polymorphs, along with a single-crystal-to-single-crystal transition between 106 and 124 °C. This structural transformation induces macroscopic motion, characterized by a distinct snakelike deformation, as observed through polarized optical and hot-stage microscopy. The snakelike motion can be explained based on the cell/supercell relation between the two crystal phases and the molecule-to-molecule mapping of the transition. Nanoindentation studies demonstrate notable variations in mechanical properties between the two phases, with significant differences in hardness and elastic modulus. Partially transformed single-crystal samples, in which the two phases coexist along with an interphase region, can be obtained by quenching the transition at room temperature. This has allowed a remarkable characterization of the interphase region by space-resolved Raman spectroscopy. The results are consistent with a model in which the interphase region, about 7 μm long, is formed by domains filled with one or the other of the two structures. In moving across the interphase, it is the concentration of daughter phase domains and of parent phase domains that regularly increases and decreases, respectively.

## Graphical abstract

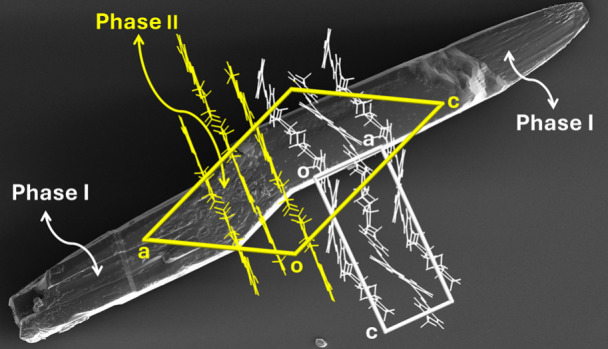

## Introduction

The concept of crystal as a “supermolecule” is generally credited to Dunitz,^1^ but it was proposed long before.^2^ This analogy has proven to be very fruitful in the development of Crystal Engineering.^3^ In molecules, atoms are bonded to each other by strong chemical bonds. In crystals, molecules are bonded to each other by weaker intermolecular bonds. In a molecular normal mode of vibration, all atoms of the molecule oscillate with the same frequency and with amplitudes and phases determined by the symmetry point group of the unperturbed molecule (in the harmonic approximation).^4^ In a low frequency normal mode of a crystal, all molecules in the crystal undergo a collective and cooperative oscillation with amplitude and phases depending on the point symmetry group of the crystal (crystal class).^5^ Molecules undergo chemical reactions in which some chemical bonds are broken and new are formed. In a crystal-supermolecule, intermolecular bonds can be broken and new can be established as well: this is exactly what happens in a crystal–crystal transition. Crystal–crystal transitions can be classified according to different criteria.^6^ A classification widely used for phase transitions in molecular crystals is between reconstructive and displacive.^6^ In reconstructive transitions there are relevant differences in the structures of the two phases and a macroscopic single crystal of the parent phase is transformed in a macroscopic specimen of the daughter phase that keeps shape and dimensions, but it is no longer a single crystal, being formed instead by a loose mosaic of many small crystals of the new phase, randomly oriented with respect to each other. In a (topotactic) displacive transition, the structural differences between the phases are minor and there is a well-defined relation between the orientation of the crystal axes before and after the transition.^7^ In this case, one single crystal of the initial phase is transformed in one single crystal of the new phase, and the transition is named single-crystal-to-single-crystal (SCSC).^8^ SCSC transitions have gained increased interest in the last years owing to the development of mechanically responsive or dynamic crystals (DCs).^9–12^ In fact, the concerted, collective and ordered movements of molecules during a SCSC transition can lead to amplification of individual molecular motions resulting in macroscopic motility.^13^ SCSC transitions are also at the root of superelastic and superplastic behavior of organic molecular crystals.^14–16^ Depending on the features of the transition, the dynamic effects can be regular, such as crystal reshaping (expansion or shrinking) or deformation (bending or twisting), or they can be stochastic, featuring motion of the crystals by hopping, and even rapid crystal splintering and fragmentation followed by scattering of the debris.^9–12^ When the SCSC transition is very fast (a displacive martensitic transition^6^), the dynamic crystals are named thermosalient^9–12^ and are appealing for actuating applications that require fast response times (milliseconds or faster).^17–19^

There are open issues related to DCs. For instance, the relation between the dynamic effects observed and the crystal structures of the parent and daughter phases.^20–22^

Predictability of the dynamic effects and of SCSC polymorphism are other important issues.^23^ The mechanism of crystal–crystal transitions, and of SCSC transitions in particular, is another point widely debated in the literature, with respect to the issues of cooperativity and collective displacements.^6,13,20–22,24–30^

Here we report on a new dynamic crystal, 4-hydroxy-N’-(4-methylbenzylidene)­benzohydrazide-*N*-methylpyrrolidin-2-one solvate (HMBB·NMP, Figure 1A). The pure HMBB compound of this solvate,^31^ an imine derivative of 4-hydroxybenzohydrazide, is a chemical analogue of the record thermosalient (jumping) crystal 4-hydroxy-N’-(2-propylidene)­benzohydrazide described earlier.^32^ We have isolated three polymorphic forms of HMBB·NMP, and two of them are related by a SCSC phase transition occurring over a broad temperature range (106–124 °C) with visible deformation of the crystals. HMBB·NMP is a rare example of a solvate molecular crystal undergoing a SCSC transition in which solvent is preserved in the crystal structure. For this phase transition, we have been able to prepare partially transformed crystals frozen at room temperature, in which the two phases coexist in the same specimen. This allowed a detailed and unprecedented characterization of the interphase domain that can be very useful to understand the mechanism of SCSC transitions.

**Figure 1:**
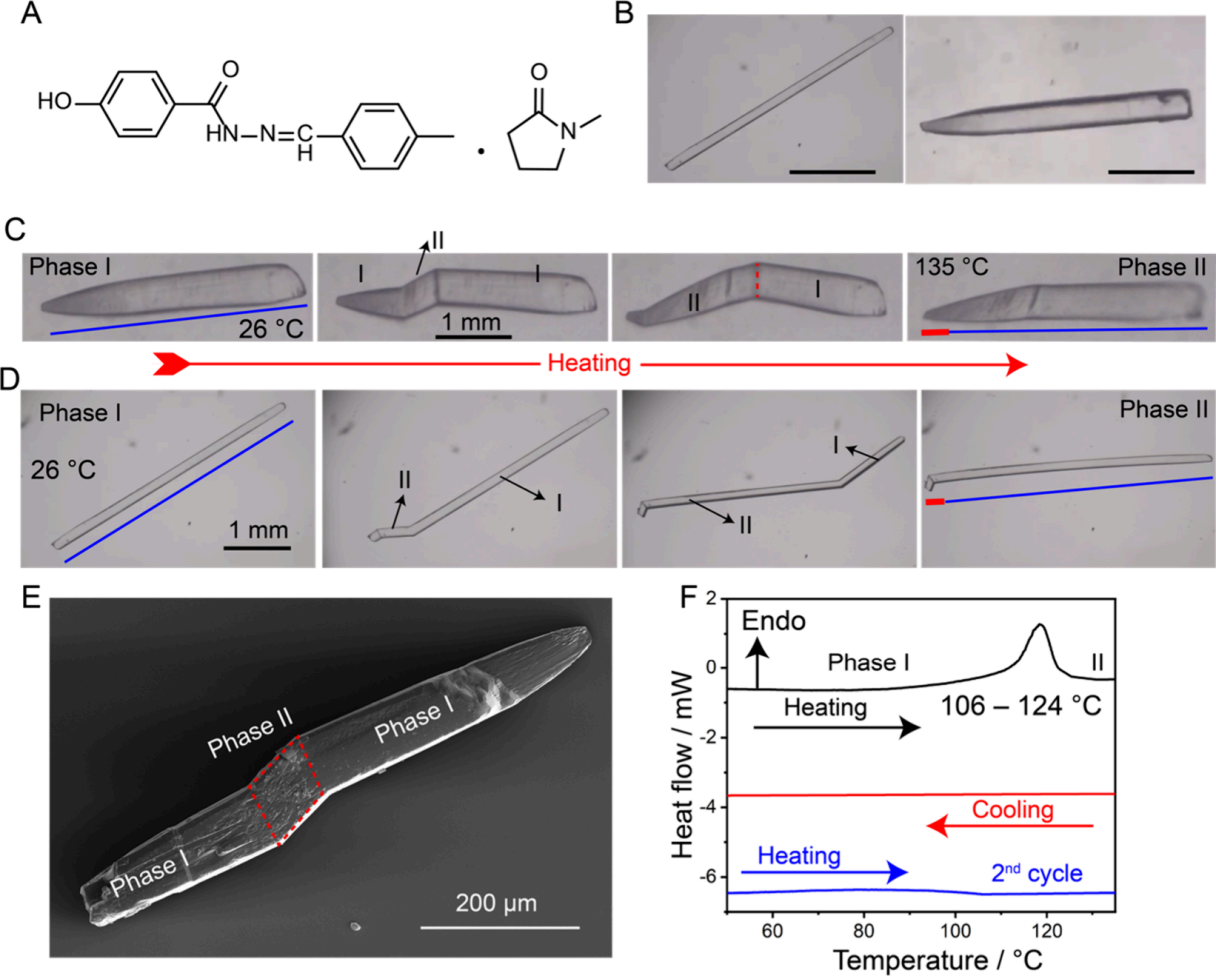
Structure and dynamic properties. (A) Chemical structure of 4-hydroxy-N’-(4-methylbenzylidene)­benzohydrazide, *N*-methylpyrrolidin-2-one solvate (HMBB·NMP). (B) Optical microscopy images of representative single crystals obtained by crystallization from solution and used for the present study. (C, D) Optical snapshots illustrating the irreversible single-crystal-to-single-crystal phase transition of elongated prismatic (C) and needle-shaped (D) crystals upon heating. In panels C and D, the blue line denotes the crystal length before the phase transition, while the red line highlights the increase in crystal length after completion of the transition. (E) Scanning electron microscopy (SEM) image of a partially transformed crystal obtained at room temperature, revealing morphological changes induced by the phase transition. (F) Differential scanning calorimetry (DSC) trace showing the thermal effects associated with the phase I to phase II transition, recorded using single crystals; the absence of a corresponding thermal event in the second heating cycle confirms the irreversible nature of the transition.

## Results and Discussion

### Polymorphism and Phase Transitions

Crystallization of HMBB·NMP afforded three polymorphs of the solvate. Large prismatic and needle shaped crystals of solvate polymorph I were obtained by heating a 20% (wt) suspension of HMBB in N-methylpirrolidin-2-one (henceforth NMP) on a hot plate until dissolution occurred, then the solution was placed in an oven at 60 °C and cooled to RT at 5 °C/h (Figure 1B). When the procedure was repeated on a more concentrated solution (33%), with the cooling starting from a higher temperature (100 °C) and performed at a slower rate (2 °C/h), prismatic crystals of a new solvated phase were obtained (polymorph III, see SI). The solvate polymorph II was obtained from phase I through an irreversible SCSC phase transition, as illustrated in Figure 1C,D. Upon heating, the original single crystals of form I undergo a cooperative structural rearrangement while preserving their overall crystallinity and external morphology. This transformation is accompanied by a pronounced anisotropic change in crystal dimensions, most notably an increase in crystal length, indicating a martensitic-like behavior rather than dissolution–recrystallization. Importantly, the crystals do not revert to their original form upon cooling, confirming the irreversible nature of the transition. Optical microscopy measurements performed on multiple crystals reveal a reproducible increase in crystal length during the phase transition, with an average elongation of approximately 8–10% after completion of the transformation (Figure 1C,D). This anisotropic strain is accommodated differently depending on crystal habit: elongated prismatic crystals frequently develop visible cracks following the transition, whereas needle-shaped crystals exhibit no observable deterioration, suggesting more efficient strain relaxation in the latter morphology. To further understand the structural changes associated with this transformation and strain accommodation, the crystal structures of the three solvate polymorphs involved were unequivocally determined by single-crystal X-ray diffraction (Table S1 in Supporting Information), while the structure of the unsolvated phase has been reported previously (ref 31).

### Characterization of the Phase Transition

In addition to the dimensional changes, both prismatic and needle-shaped HMBB crystals exhibited a distinctive snakelike motion during the phase I to phase II transition. Hot-stage polarized optical microscopy (POM) performed at a heating rate of 10 °C/min captured this behavior in real time (Figure 1C,D and Movies 1 to 5 in SI). The motion reflects the cooperative structural rearrangement within the crystals, illustrating their dynamic mechanical response and flexibility as they undergo the irreversible phase transformation. The surface morphology during the phase transition was examined using scanning electron microscopy (SEM) on a partially converted crystal (Figure 1E). This analysis provided detailed insights into the structural changes occurring during the transition from phase I to phase II and shows the clear domains, complementing the optical observations captured through hot stage POM. The phase transition was observed in the temperature range 106–124 °C depending on the morphological features of the crystal specimen (thickness, surface smoothness, regularity).

This phase transition was further confirmed by differential scanning calorimetry (DSC) analysis (Figure 1F). The heating run exhibits a sharp endothermic signal centered at 118 °C with an enthalpy change of ΔH = 2.2 kJ/mol, corresponding to the irreversible SCSC transition from phase I to phase II. The DSC data also demonstrate that the transition is monotropic, as the event is no longer observed upon cooling or during a second heating cycle, confirming the nonreversible nature of the transformation. In addition, a comparison with thermogravimetric analysis (TGA, see Figure S5 in Supporting Information) indicates that the observed thermal event is not associated with solvent loss or decomposition, further supporting that the endotherm arises solely from the phase transition. The TGA analysis reveals that crystals of HMBB solvate phase I show a first weight loss (29%) in the range 130–150 °C that clearly corresponds to the loss of NMP (the theoretical value is 28%). After solvent removal, the sample is stable up to melting point (275 °C). Heating prismatic crystals of phase I to the transition temperature is accompanied by an extraordinary and readily visible reshaping (Figure 1C,D). The phase transition appears as a new domain, which grows toward the ends of the crystal. The time it takes to complete the phase transition depends on the morphology and the intrinsic quality and perfection of the crystals.^25^ Some thick crystals transform in 10–15 s (Movie S1 in SI), while some very thin crystals transform in less than a second (Movie S2 in SI). A remarkable feature of the HMBB·NMP system is that the shape transformation of the crystals during the transition can also be influenced by the way they are positioned or physically constrained on the substrate and the specific crystal face in contact with the surface. These factors can affect how the crystals accommodate the strain and undergo dimensional changes during the transformation. If thin, long crystals are constrained at one end, they rotate for about 30° around the fixed tip (Movie S3 in SI). This motion is accomplished through lateral shifts of consecutive slices of the crystal in a slithering fashion. If both ends of the crystal are fixed, the deformation propagates along the crystal, while it remains in its original position (Movie S4 in SI). In other cases, for a crystal unconstrained, the transition starts in the middle of the crystal and propagates toward the tips, and the crystal arches over the substrate at first and eventually flips (Movie S5 in SI). In some specimens, the transition occurred very rapidly (milliseconds), with the crystal undergoing rapid movements.

### Structures of Polymorphs

The crystals of form I are orthorhombic, space group *P*2_1_2_1_2_1_, with lattice parameters (at – 100 °C) *a* = 7.3790(12) Å, *b* = 14.380(2) Å, *c* = 17.192(3) Å, *V* = 1824.2(5) Å^3^, Z = 4. In the structure (Figure 2A,B), one imine molecule and one NMP molecule are crystallographically independent.

**Figure 2:**
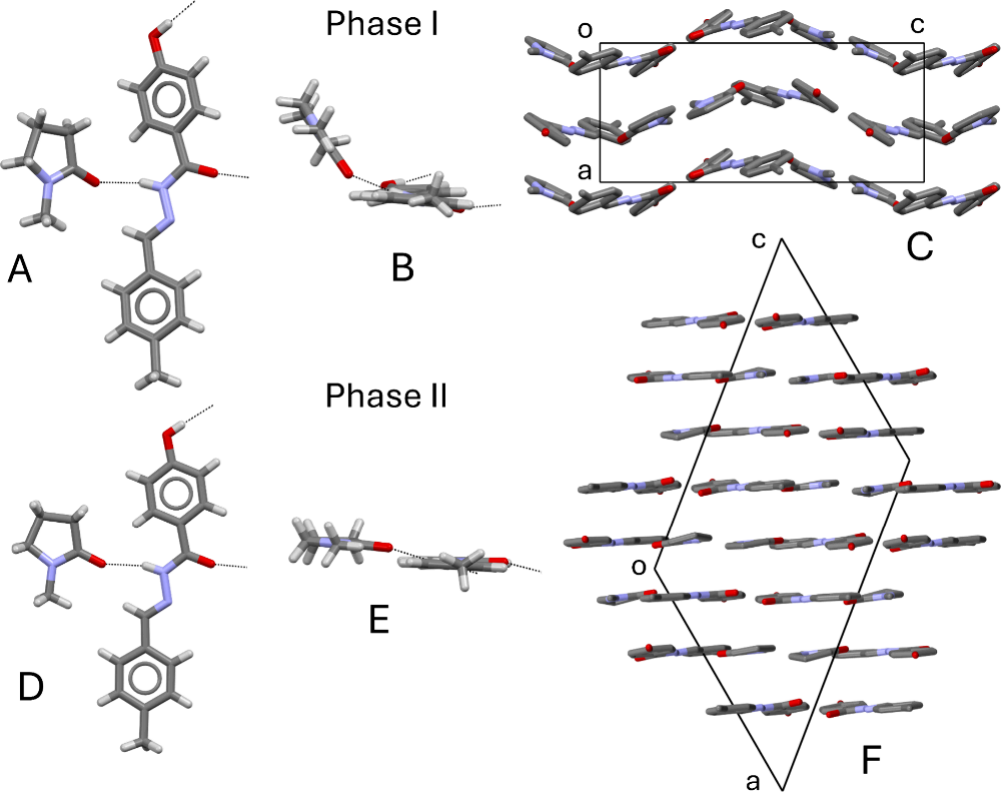
Relevant structural features of phases I and II. (A) face view of an imine molecule H-bonded to a NMP molecule in phase I; (B) the same couple of molecules of A viewed down the long molecular axis; (C) crystal packing of phase I viewed down **b** (H atoms omitted for clarity); (D) face view of an imine molecule H-bonded to a NMP molecule in phase II in which only one split position of the disordered NMP molecule is shown; (E) the same couple of molecules of D viewed down the long molecular axis; (F) crystal packing of phase II viewed down **b** (H atoms omitted for clarity).

Chains running parallel to **b** are formed by strong hydrogen bonds between the phenolic O–H group and the imine carbonyl oxygen. The chains are wrapped around 2_1_ screw axes. H-bonds are also formed between amide N–H group and carbonyl O of the NMP molecule. So, the H-bonded chains are laterally decorated with NMP molecules and ribbons are formed. This topology of hydrogen bonding is retained in the three polymorphs (see Figure S8 in Supporting Information). The basic conformational feature of the imine molecule, i. e. the O–H phenolic hydrogen pointing in the same direction as the amide C = O carbonyl, is also consistently found in the three polymorphs. The H-bonded chains with the lateral H-bonded NMP molecules form ribbons that are arranged in undulated layers, which are stacked along the crystallographic axis **a** (Figure 2C). The undulation of the layers is mainly due to deviation from planarity of the NMP and imine molecules, as shown in Figure 2B.

Phase II is monoclinic, space group *P*2_1_/*c*, with lattice parameters (at – 100 °C) *a* = 15.384(6) Å, *b* = 14.307(5) Å, *c* = 21.295(8) Å, β = 129.01(2)°, *V* = 3642(2) Å^3^, Z = 8. There are two crystallographically independent imine molecules, related by a pseudocenter at (0.25**a**, 0**b**, 0.5**c**) and two (disordered) NMP molecules. The NMP molecules form hydrogen bond with N–H of the imine molecules as in phase I but, in phase II, the planes of the imine and NMP molecules are almost parallel, Figure 2D,E. So, the ribbons are arranged in molecular layers that are not undulated but essentially flat, and are stacked along the **a**-**c** diagonal, as shown in Figure 2F.

The crystal structure of form III, which is not immediately relevant to the dynamic properties of HMBB NMP, is triclinic, space group *P*1̅, with lattice parameters (−100 °C) *a* = 7.786(3) Å, *b* = 14.321(3) Å, *c* = 16.686(3) Å, α = 91.048(16)°, β = 96.35(2)°, γ = 99.57(3)°, *V* = 1822.1(8) Å^3^, Z = 4 (see SI). Two imine molecules and two disordered NMP molecules are crystallographically independent. Metrically, the unit cell of phase III is similar to phase I, while the arrangement of NMP molecules is like phase II. Heating crystals of phase III results in loss of NMP solvent molecules and formation of pure HMBB, without any solid–solid transition.

### Molecular Mapping of the Phase Transition between Phases I and II

The SCSC nature of the transition between forms I and II suggests a structural relationship between the unit cells of the two phases.^13,21,22^ In fact, by suitable matrix transformations, it is possible to find for both the orthorhombic phase I and the monoclinic phase II a common monoclinic supercell. The matrix transformation of unit cells of phase I and II leading to the supercell are described in detail in SI.

Half of the 2_1_ screw axes parallel to **b** of the orthorhombic phase, namely those related to the formation of the H-bonded chains, are retained as crystallographic symmetry elements in the monoclinic *P*2_1_/*c* phase II (see Figure S9A in Supporting Information). With the unit cell transformation at hand, it is possible to come up with a molecule-to-molecule mapping of the phase transition and its effect on the crystal shape. At microscopic level, there is close metric similarity between the position of molecules in the two phases, as is clearly evidenced in the superposition of the two polymorphs shown in Figure 3 (see also Figure S9B in Supporting Information).

**Figure 3:**
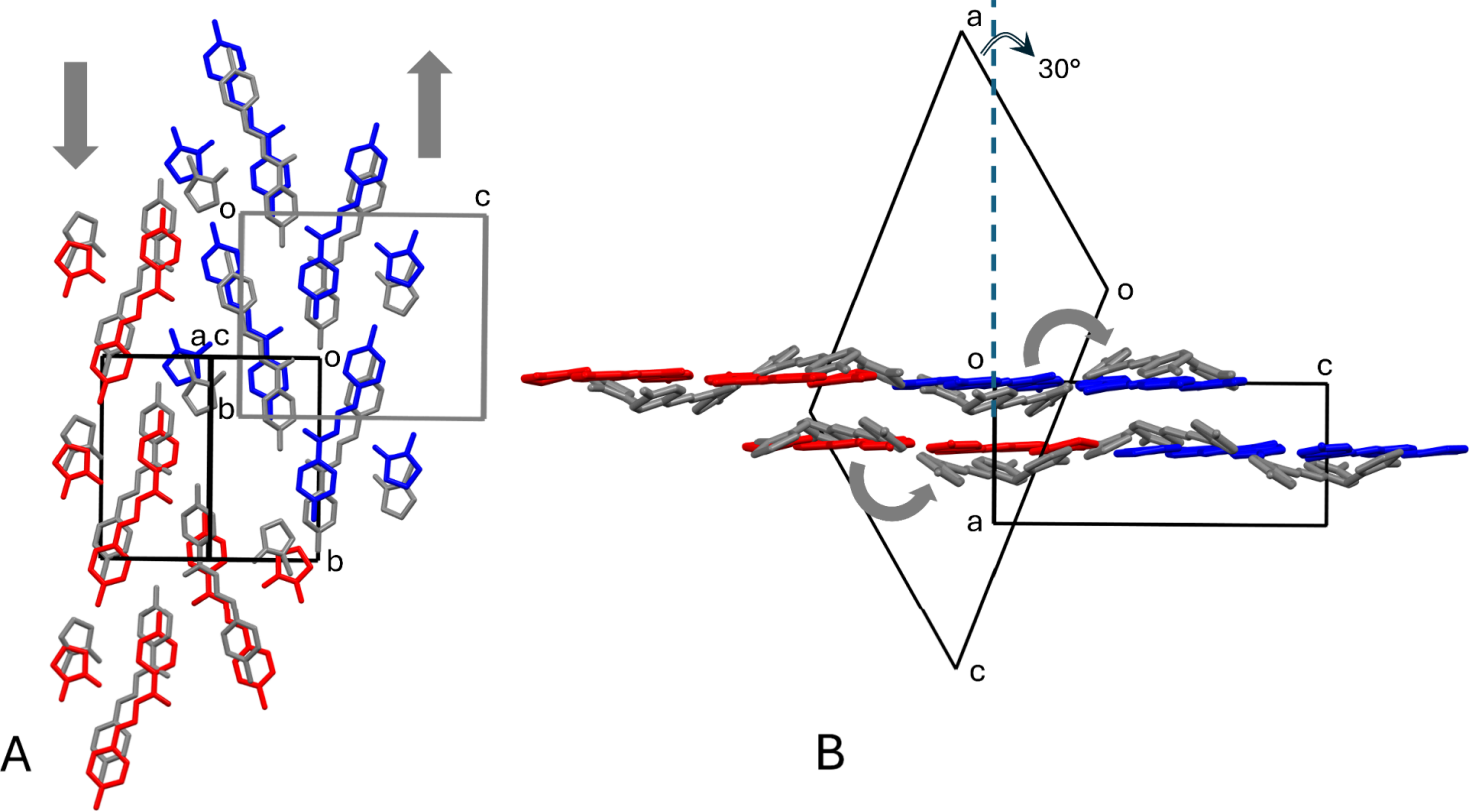
Some relevant structural features for the analysis of the transition from phase I to II. (A) Superposition of phase I (in gray) and of phase II, whose independent molecules are shown in red and blue, down the common direction of axis **a** of phase I and axis **a**-**c** of phase II. The gray arrows indicate the sense of translation of molecules of phase I in going to phase II; (B) View down the common axis **b** of superposition of phase I and phase II. The gray arrows indicate the sense of rotation of imine molecules of phase I in going to phase II. In A and B, the unit cell of phase I and of phase II are oriented according to the transformation matrices described in SI and in such a way that the 2_1_ axes parallel to **b** of phase I involved in the formation of H-bonded chains are kept in phase II. The angle between axis **a** of the orthorhombic and monoclinic cells is also evidenced in B. Only one split position of the disordered NMP molecule of phase II is shown. H atoms omitted for clarity. Some NMP molecules are omitted in A for clarity.

The superimposition is achieved through a translation, in opposite directions, of molecules of phase I along the 2_1_ axes parallel to **b**, that are kept in polymorph II (Figure 3A, see also Figure S9 in Supporting Information). The transformation from phase I to II also requires the (local) rotation of solvent NMP molecules, that in phase II are coplanar with the imine molecules. During this rotation, there is enough space for NMP molecules to also flip around the direction of the H-bond. In fact, in phase II they are statistically disordered in two split positions (Figure S7 in Supporting Information). So, solvent NMP molecules play a role of both “lubricant” and “cushion” between adjacent ribbons during the transition, helping to preserve crystal integrity. Some rotations around **b** of the imine molecules are also involved during the transition. As shown in Figure 3B, imine molecules of phase I that end up in red or blue independent molecules of phase II, rotate by the same angle but in different senses. This difference, coupled with the different sense of translation along **b** (Figure 3A) is, perhaps, the reason why there are two independent molecules in phase II, out of one in phase I. It may seem surprising that a SCSC transition occurs between an acentric (phase I) and a centrosymmetric (phase II) crystal structure. However, it should be noted that the acentric phase I has *P*2_1_2_1_2_1_ as the space group. This latter is not a polar space group^31^ and so the structure of phase I already has the up and down arrangement of molecules as requested by a centrosymmetric crystal structure. At the macroscopic level, crystals of phase I are prisms elongated in the **a** direction (Figure 1B, see also Figure S6 in Supporting Information), which is also the direction of stacking of the undulated layers of Figure 1C. After the transition to phase II, which involves the formation of the planar layers of Figure 2F, the prismatic crystals now obtained are elongated in the **a** direction of the monoclinic cell (see Figure S6 in Supporting Information). So, in the *locus* in which the transition starts, the front of phase II grows and advances along **a**, therefore forming an angle which is exactly defined because it is the angle between the directions of **a** in the orthorhombic and monoclinic lattices (Figure 3B). This angle, which is constantly found in the crystals undergoing the transition (Figure 1C–E), is basically responsible for the snakelike motion observed during the phase transformation.

### Characterization of the Interphase Region

The possibility of obtaining partially converted crystals frozen at room temperature, Figure 1E, suggests that each small part of a crystal specimen is subject to an activation barrier (multistep mechanism); this is at variance with thermosalient transitions that are very fast (displacive martensitic) and for which partially transformed samples are generally not reported. Partially transformed samples are also ideal for an accurate analysis of the interphase region because they embody a snapshot of the sample while converting from one phase to the other. We have performed a Raman analysis of a partially transformed sample, Figure 4A, by scanning the crystal along a line crossing the transition region, Figure 4B. The spectra were recorded at consecutive steps of 0.8 μm (see SI) which is also the size of the laser spot. The analysis was performed in the low wavenumber region (10–110 cm^–1^) which is most sensitive to subtle differences in the packing.

**Figure 4:**
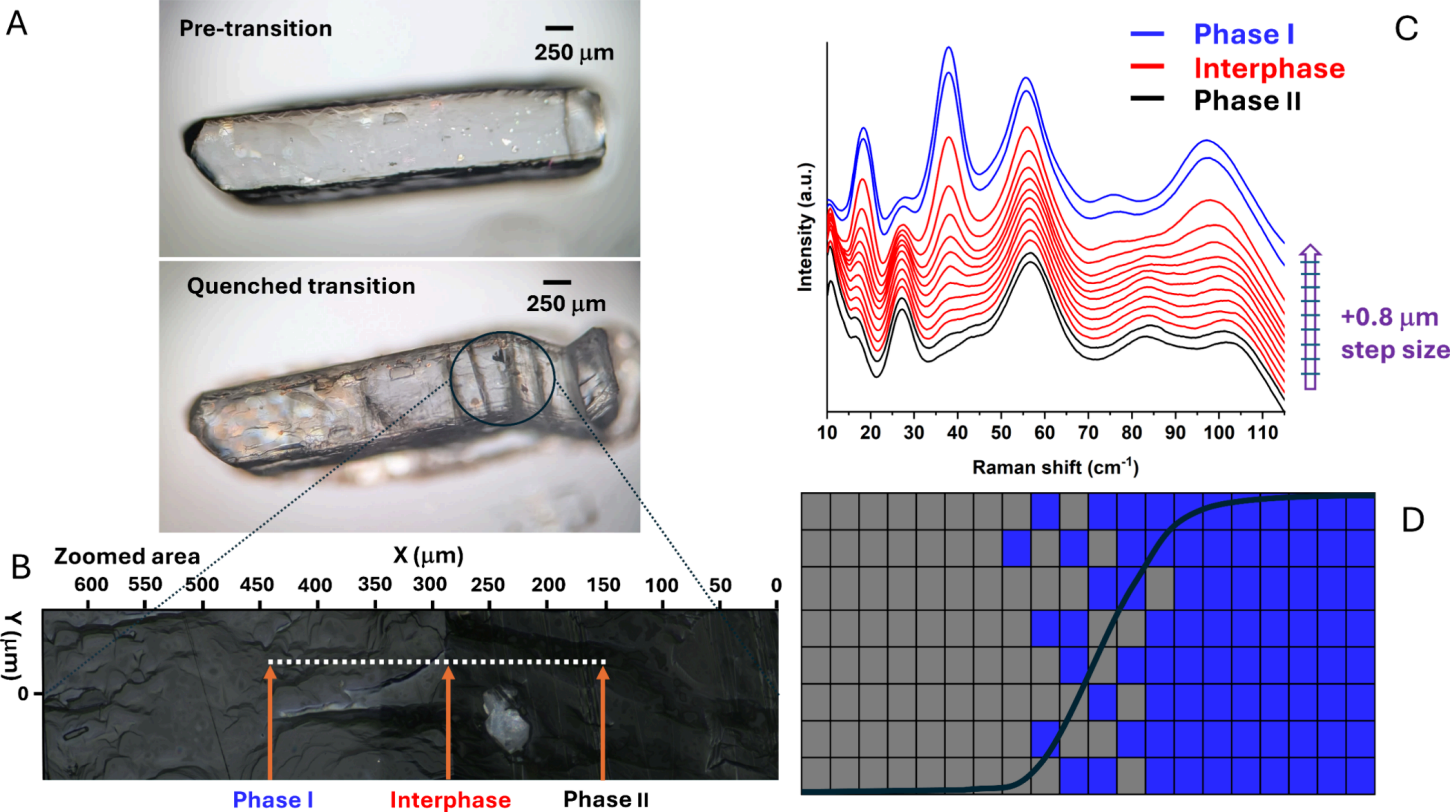
Characterization of the interphase region in a partially transformed sample. (A) Microscope images of a single crystal in phase I (up) and of the same crystal partially transformed in phase II (down), with the interphase region encircled. (B) Zoomed image of the interphase region with indication of the scan line. (C) Raman spectra recorded at 0.8 μm steps across the interphase region. (D) Possible sketch of the interphase region. Gray color indicates phase II, blue phase I. A sigmoid curve is superimposed as a guide to the eye.

In Figure 4C the whole spectra are reported at different distances along the line of scan. In this set of spectra, a gradual change of the Raman spectrum is observed in going from phase II to phase I through the interphase region, as also reported in a similar analysis.^30^ This would qualify the interphase region as one in which there is a continuous rather than clear-cut change. The size of the interphase can be evaluated with reference to the mode at 37.60 cm^–1^ of phase I (calculated at 39.25 cm^–1^, see Table S5 in Supporting Information), which is absent in phase II, and the mode at 55.77 cm^–1^ (calculated at 57.38 cm^–1^), Figure 4C. The mode at 39.25 cm^–1^ is primarily an NMP rotational motion in the (**b**,**c**) plane (see Movie S6 in SI), while the mode at 57.38 cm^–1^ is basically a bending vibration of HMBB and NMP molecules with respect to each other (see Movie S7 in SI). Both modes, together with the mode at 19.22 cm^–1^ (calculated at 20.19 cm^–1^ and involving combined rotation around **b** of HMBB and NMP molecules, see Movie S8 in SI) embody motions such as those previously described in the molecular mapping of the transition between phases I and II. As also shown in Figure S12 of SI, between the first change of the intensity ratio of the mode at 37.60 cm^–1^ with the mode at 55.77 cm^–1^ and its disappearance, there are nine steps, so one can evaluate Δ*s* = 9 · 0.8 *μm* = 7.2 *μm* for the size of the interface. So, the linear spanning of the interphase region can be estimated to be 7 μm. Considering that for phase I it is a = 7.3790 Å, it follows that the interphase region is about 10^4^ unit cell long. To put on a quantitative basis the gradual change of structure across the interphase region, we have evaluated, for each normalized spectrum, the ratio *R* of the intensity of the peak at 37.60 cm^–1^ to that of the peak at 55.70 cm^–1^. The plot of *R* as a function of the scan distance is shown in Figure S13 of SI. The plot has a sigmoidal (logistic) shape that is typical of growth processes undergoing saturation. This suggests a model in which, within the interphase region, the domains (cells) of the regular array forming the crystal are filled either by the parent structure or by the daughter one, Figure 4D. In moving across the interphase region, it is the concentration of domains filled with each structure that does change, ranging from (*x*_*I*_ = 0, *x*_*II*_ = 1) to (*x*_*I*_ = 1, *x*_*II*_ = 0). The metric matching between the two lattices and the presence of a common supercell (see Figure S9B in Supporting Information) allow the monolithic seamless nature of the crystal specimen to be preserved in the interphase region also. This picture is further supported by the finding that the sequence of experimental spectra of Figure 4C can be well reproduced by summing the experimental spectra of pure phases I and II each multiplied by the proper mole fraction (see Figure S14 in Supporting Information). A similar model has been proposed for homogeneous SCSC solid-state reactions.^33^

### Mechanical Properties

The relevant dynamic behavior associated with the irreversible SCSC transition can suggest variation in the mechanical properties of the two forms. In particular, properties such as hardness, elasticity, and fracture behavior may differ significantly between the two phases, also depending on the crystal morphology and the direction of applied stress. The mechanical properties such as stiffness (E) and hardness (H) of crystals of both HMBB·NMP phases were determined by nanoindentation on their physically accessible faces after identifying the crystallographic faces. The accessible crystal faces that could be indented were matched with Bravais–Friedel–Donnay–Harker (BFDH) morphology that was reconstructed from their crystal structures for both phases I and II. The load–displacement curves recorded at a selected penetration depths on (011)/(01̅1̅) and (011)/(01̅1̅) for phases I and II respectively are shown in Figure 5A,B.

**Figure 5:**
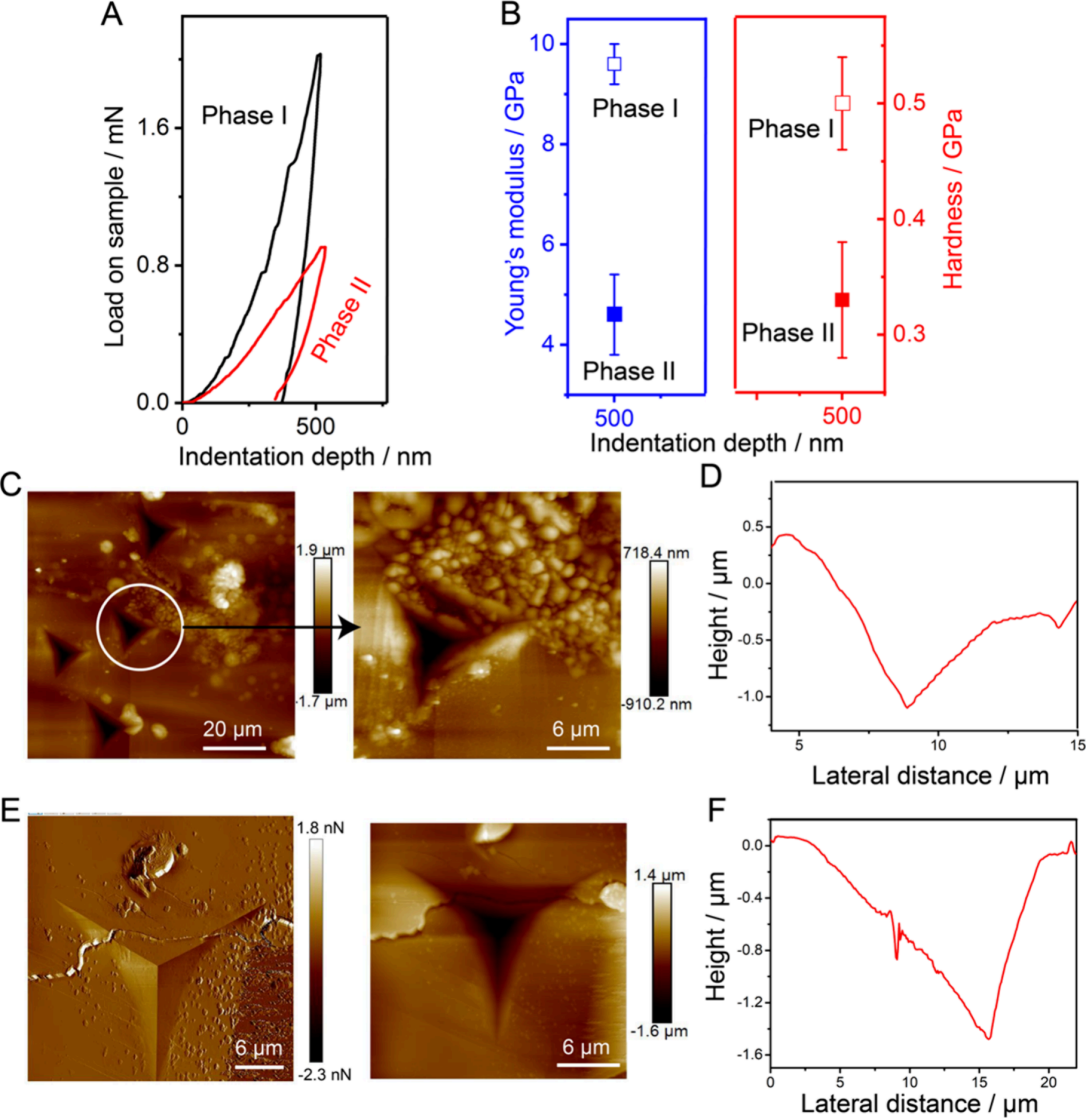
Mechanical properties and AFM analysis of phase I and phase II crystals. (A) Load–depth curves recorded separately for phase I and phase II crystals on their (011)/(01̅1̅) faces at a selected penetration depth, illustrating the differences in mechanical response between the two phases. (B) Young’s modulus (E) and hardness (H) values calculated from curves similar to those shown in (A). Error bars represent the standard deviations obtained from 10–15 independent indents at each indentation depth, reflecting the reproducibility of the measurements. (C,E) Representative AFM topography images of the indent impressions on phase I and II crystals, highlighting the uniformity of the indents and the absence of material pileup around the edges, which indicates minimal plastic deformation during indentation. (D,F) Height profile corresponding to the indent shown in (C,E), providing a quantitative view of the depth and morphology of the indentation and further confirming the mechanical response of the crystal surface.

The residual impressions were imaged by atomic force microscopy (AFM) and no significant material pile-up was observed along the edges of the indenter impressions as shown in Figure 5C,E. The average elastic modulus and hardness (H) of phase I on its (011)/(01̅1̅) face was found to be E= 9.6 ± 0.4 GPa and H = 0.5 ± 0.04 GPa for 500 nm indentation depth for a total of 14 indents. The modulus of phase I on its (011)/(01̅1̅) face is relatively higher compared to those of some mechanically responsive crystals.^34–36^ The fully transformed crystal of phase II after heating was used for the nanoindentation experiments at room temperature. The average elastic modulus of phase II on its (011)/(01̅1̅) face was E= 4.6 ± 0.8 GPa and hardness (H) = 0.33 ± 0.05 GPa for 500 nm indentation depth for a total of 12 indents. Indeed, the elastic modulus of phase I is approximately twice that of phase II, highlighting how the SCSC transformation leads to a pronounced reduction in stiffness. These results indicate that phase II is significantly softer than phase I. Based on recent literature,^34–36^ phase II can be classified as a relatively soft material, further emphasizing the strong correlation between crystallographic packing, phase transition, and mechanical response.

The drop of Young's modulus coupled with the concentration gradient model of the interphase domain can help in understanding why the monolithic nature of the crystal specimens is preserved during the SCSC transition, without developing brittle fractures or shattering, in spite of the significant bending (snakelike motion) and anisotropic strain (8–10% elongation). This is particularly remarkable if we consider the case of crystals that are physically constrained during the transition at one or both ends (see Movies S3 and S4 in SI).

## Conclusion

This study provides valuable insights into the dynamic and mechanical properties of HMBB·NMP and its polymorphs. The snakelike motion observed during the SCSC transition is a clear and direct macroscopic manifestation of the structural rearrangement at the molecular level, offering a new example of the translation of microscopic molecular motion into macroscopic body motion.^13^ The solvate nature of the HMBB·NMP system, with both the imine and NMP solvent molecules involved in the structural rearrangements, can influence the kinetic features of the transition. The results contribute to the growing body of knowledge on dynamic molecular crystals and their potential for integration into functional materials with a broad range of technological applications. The space-resolved Raman analysis performed on the interphase region in frozen biphasic crystals of HMBB·NMP can provide valuable information on some mechanistic aspects of SCSC transitions. These transitions have singular features within the realm of crystal–crystal transitions. For instance, no new surface is formed after an SCSC transition: the external faces of the parent crystal are kept as external faces of the daughter crystal. This point is relevant with respect to the classic nucleation/growth mechanism of first order phase transitions which is based on the interplay between volume free energy and surface free energy.^6^ Our analysis/model of the interphase region suggests that the SCSC transition takes place within single unit cells, without creation of new surfaces. The other evident singular feature of SCSC transitions is cooperativity, namely cooperative microscopic molecular motions stretching over macroscopic distances (the whole size of the single crystal). In the case of thermosalient crystals, in which the SCSC transition is displacive martensitic, recent studies suggest that the transition is activated by low frequency lattice vibrations,^20,22,37–41^ and so cooperativity would naturally result from the oscillations of the whole crystal-supermolecule (a mechanical transition^22^). In the present case, cooperativity is related to the propagation of the transition between neighboring cells within the interphase region (Figure 4D) and this could be achieved also by different mechanisms (avalanche/domino effects).

## Supplementary Material



















## Notes

Transition I→II for a thick crystal, movie_1 (MP4)
Transition I→II for a needle-shaped crystal, movie_2 (MP4)
Transition I→II for a crystal with one tip fixed, movie_3 (MP4)
Transition I→II for a crystal with both tips fixed, movie_4 (MP4)
Transition I→II for a slender crystal with arching and flipping, movie_5 (MP4)
Animation of the calculated normal mode at 39.25 cm-1 for phase I, movie_6 (MP4)
Animation of the calculated normal mode at 57.38 cm-1 for phase I, movie_7 (MP4)
Animation of the calculated normal mode at 20.19 cm-1 for phase I, movie_8 (MP4)
Full description of general experimental procedures; ^1^H and ^13^C NMR spectra of HMBB; DSC and TGA analysis of HMBB·NMP; full description of the X-ray analysis; table with full crystallographic details (crystal, collection, and refinement data) of the three polymorphs; face indexing of single crystals of polymorphs I and II; description of the crystal structure of polymorph III; description of the H-bonds in the three polymorphs; description of the metric relation between polymorphs I and II; analysis of the internal motions in phase I; description of Raman measurements; computational analysis of phases I, II and III with analysis of normal modes of phase I (PDF)
