# Supplementary material for: Frozen Interphase Domain and Mechanism of the Snakelike Macroscopic Motion in a Dynamic Crystal Solvate
Source: J Am Chem Soc. 2026 Apr 27;148(17):18162–70. doi: 10.1021/jacs.6c02665 (PMC13159555; doi:10.1021/jacs.6c02665)
Supplement: Supplementary file 9 [file ja6c02665_si_009.pdf]

Supporting Information file

Frozen Interphase Domain and Mechanism of the Snakelike Macroscopic Motion in a Dynamic Crystal Solvate

Emmanuele Parisi[a§], Fabio Borbone[b§], Elena Simone[a], Luca Catalano[c], Durga Prasad Karothu[d], Sanjit Manohar Majhi[d], Ejaz Ahmed[e], Salvatore Zarrella[f], Timothy M. Korter[f], Roberto Centore[b]*

[a] Department of Applied Science and Technology, Politecnico of Turin, I-10129 Turin, Italy.
[b] Department of Chemical Sciences, University of Naples Federico II, Via Cintia, I-80126 Naples, Italy.
[c] Dynamic Molecular Materials Laboratory, Department of Life Sciences, University of Modena and Reggio Emilia, Via G. Campi 103, 41125 Modena, Italy.
[d] Center for Smart Engineering Materials, New York University Abu Dhabi, PO Box, 129188 Abu Dhabi, United Arab Emirates.
[e] Smart Materials Lab, New York University Abu Dhabi, P.O. Box, 129188 Abu Dhabi, United Arab Emirates.
[f] Department of Chemistry, Syracuse University, 111 College Place, Syracuse, New York, U. S. A. 13244-4100.
[§] These authors contributed equally to this work.

1.Experimental Part……p.2
2. NMR spectra……p.2
3. DSC and TGA analysis……p.3
4. X-ray analysis……p.5
    4.1. Experimental data and refinement details……p.5
    4.2. Morphological properties of crystals of phases I and II……p.6
    4.3. Crystal structure of phase III……p.7
    4.4. H-bonding in phases I, II and III……p.8
    4.5. Metric and symmetry relations between phases I and II……p.9
    4.6 Analysis of internal motions in phase I by X-ray diffraction data……p.10
5. Raman measurements……p.14
    5.1 Experimental details……p.14
    5.2 Discussion……p.14
6. Nanoindentation measurements……p.16
7. Computational analysis……p. 17
8. References……p.20

1.Experimental Part

General. All reagents were analytical grade and were used without further purification. HMBB was prepared according to a procedure already described.[1] Transition points were determined by temperature controlled optical microscopy (Zeiss Axioskop polarizing microscope equipped with a Mettler Toledo HS82 Hot Stage and HS 1 hot stage controller) and DSC analysis (Mettler Toledo DSC 1 apparatus, N_2 atmosphere, scanning rate 10 K/min on both the heating and cooling cycles). TGA was performed with a PerkinElmer TGA 4000 apparatus at 10 K/min heating rate under flowing N_2 atmosphere. NMR spectra were recorded with Bruker spectrometer operating at 500 MHz, in DMSO-d_6. FESEM FEI Nova NanoSEM 450 instrument was used to image crystals with Scanning Electron Microscopy (SEM). The crystals were attached to a carbon tape and sputtered in a DentonVacuum Desk V HP sputterer using an alloy of gold/palladium target prior to observation.

2. NMR spectra

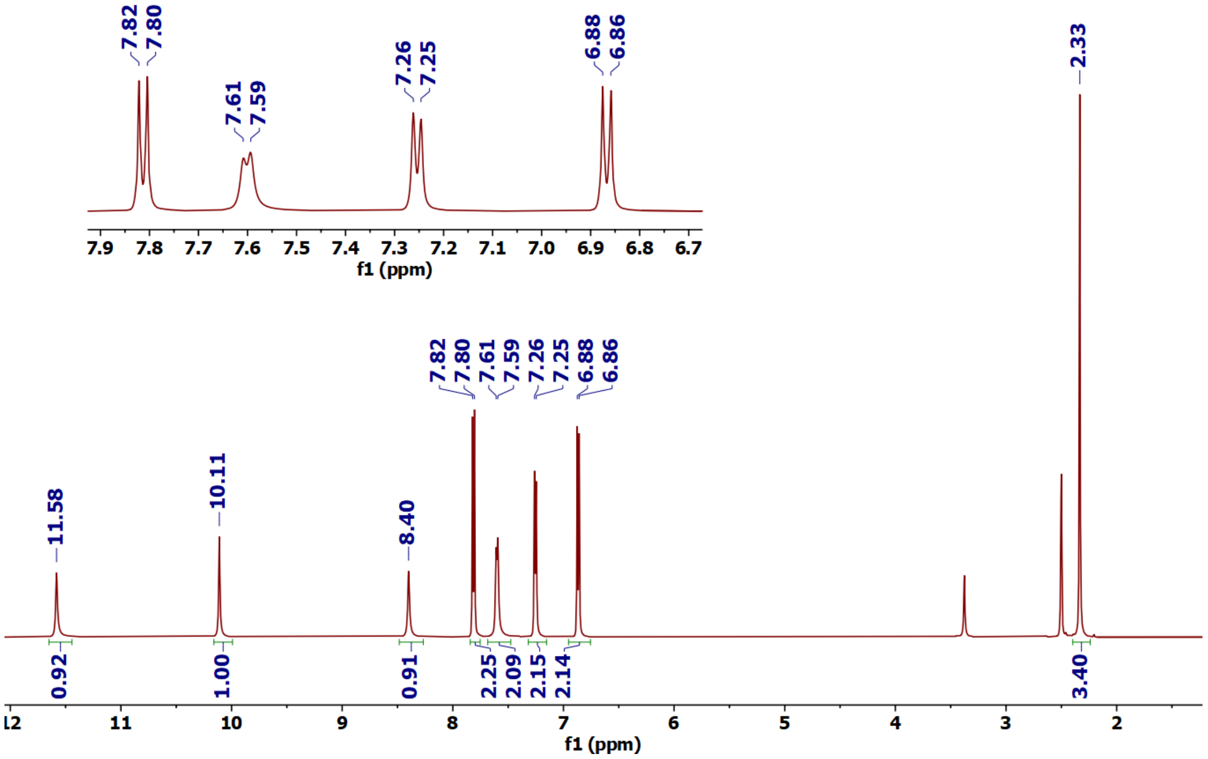

Fig. S1. ^{1}H NMR spectrum of HMBB (500 MHz, DMSO-d_6). δ 11.58 (1H s), 10.11 (1H s), 8.40 (1H s), 7.82 (2H d), 7.61 (2H d), 7.26 (2H d), 6.88 (2H d), 2.33 (3H s). The signals at 2.51 ppm and at 3.35 ppm are due to the solvent and water, respectively.

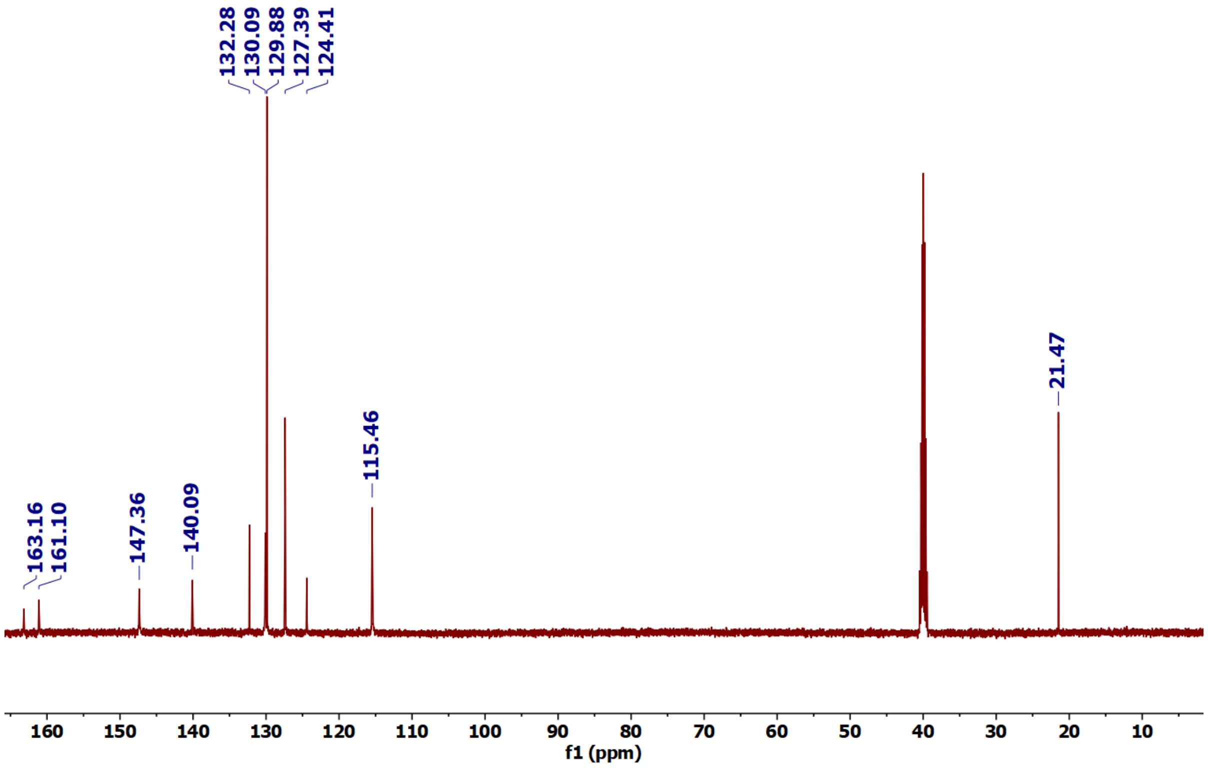

Fig. S2. ^{13}C NMR spectrum of HMBB (100 MHz, DMSO-d_6). δ 163.16, 161.10, 147.36, 140.09, 132.28, 130.09, 129.88, 127.39, 124.41, 115.46, 21.47. The signals nearly 40 ppm are due to the solvent.

3.DSC and TGA analysis

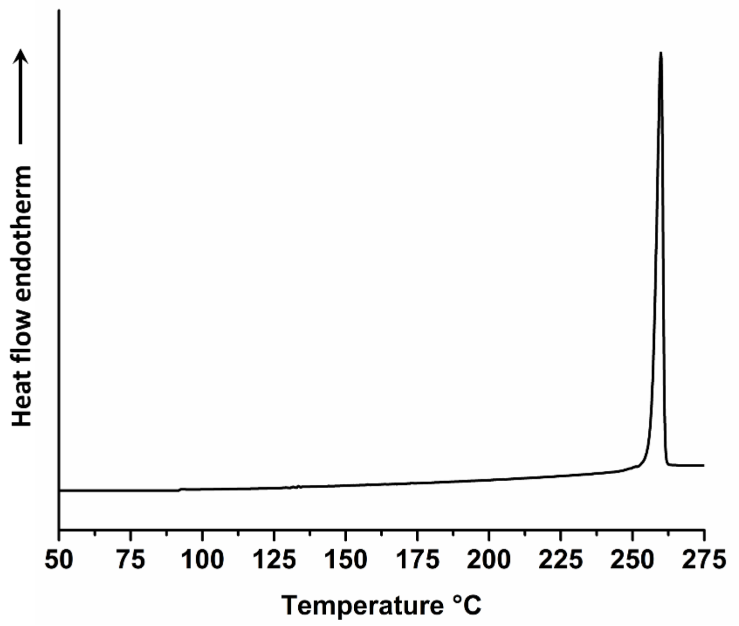

Fig. S3. DSC thermogram, on heating, of pure HMBB. Heating rate 10 K/min under flowing N_2.

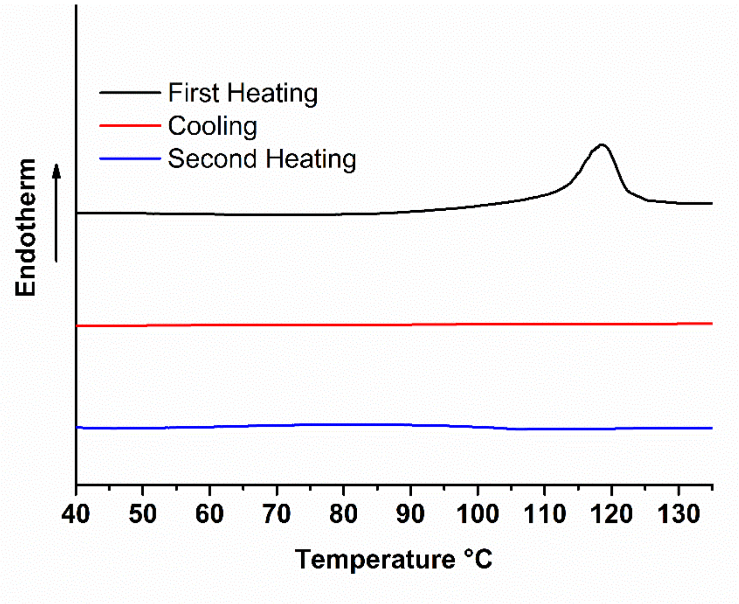

Fig. S4. DSC thermogram, on heating, of HMBB solvate polymorph I. Heating rate 10 K/min under flowing N_2.

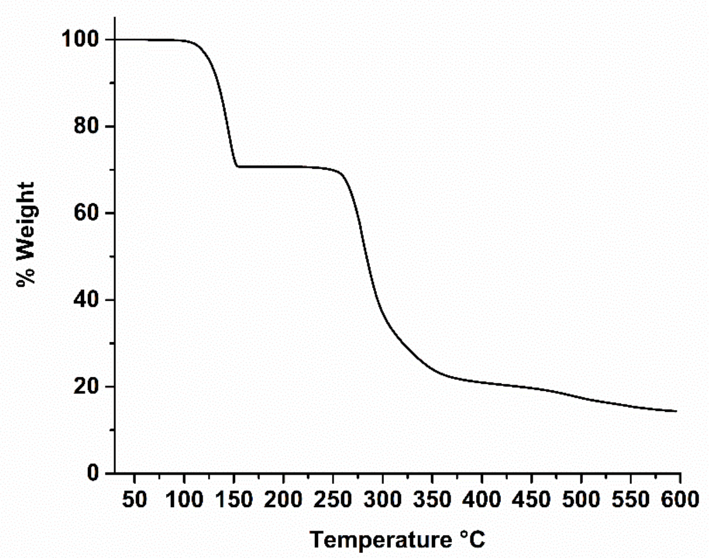

Fig. S5. TGA analysis of HMBB solvate polymorph I. Heating rate 10 K/min under flowing N_2.

4.X-ray analysis

4.1 Experimental data and refinement details

All data for crystal structure determinations were measured on a Bruker-Nonius Kappa CCD diffractometer equipped with an Oxford Cryostream 700 apparatus, using graphite monochromated MoKα radiation (λ = 0.71073 Å). Reduction of data and semiempirical absorption correction were done using the SADABS program.[2] The structures were solved by direct methods (SIR97 program[3]) and refined by the full-matrix least-squares method on F^2 using the SHELXL-2019/2 program[4] with the aid of the program WinGX.[5] H atoms bonded to C were placed in calculated positions and refined by the riding model; H atoms bonded to N and O were clearly found in difference Fourier maps as the first maxima, and their coordinates were refined. For all H atoms, U_{iso} =1.2 × U_{eq} of the carrier atom was assumed. In polymorphs II and III the solvent NMP molecules are disordered. The disorder is static and was modelled with two split positions that were refined with some constraints and restraints on bond lengths and ADPs. Crystal and refinement data are summarized in Table S1. The analysis of the crystal packing was performed using the program Mercury.[6] CCDC deposition numbers 2514808, 2514809, 2514810, 2537564 contain the supplementary crystallographic data for this article.

Table S1. Crystal, collection, and refinement data for the structures described in the paper.

	Polymorph I		Polymorph II	Polymorph III
	$C_{15}H_{14}N_2O_2{\cdot}C_5H_9NO$			
M_r	353.41			
System	Orthorhombic		Monoclinic	Triclinic
Space group	$P2_12_12_1$		$P2_1/c$	$P\bar{1}$
Temp. (K)	293	173	173	173
a (Å)	7.435(3)	7.3790(12)	15.384(6)	7.786(3)
b (Å)	14.366(7)	14.380(2)	14.307(5)	14.321(3)
c (Å)	17.454(9)	17.192(3)	21.295(8)	16.686(3)
α (°)	90	90	90	91.048(16)
β (°)	90	90	129.01(2)	96.35(2)

γ (°)	90	90	90	99.57(3)
V (Å^3)	1864.3(16)	1824.2(5)	3642(2)	1822.1(8)
Z/Z'	4/1	4/1	8/2	4/2
d (g/cm^3)	1.259	1.287	1.289	1.288
λ/μ (mm^{-1})	Mo*K*α/0.09	Mo*K*α/0.09	Mo*K*α/0.09	Mo*K*α/0.09
Meas.,indep. Reflns	9746, 4012	9645, 3937	50606, 8324	20862, 8278
R_{int}	0.0319	0.059	0.090	0.064
$(\sin\theta/\lambda)_{max}$ (Å^{-1})	0.650	0.650	0.650	0.650
R[I > 2σ(I)], *wR*(all), S	0.050, 0.114, 1.07	0.048, 0.129, 1.05	0.059, 0.151, 1.06	0.056, 0.135, 1.03
Data/param/restraints	4012/243/0	3937/243/0	8324/541/96	8278/549/96
$\Delta\rho_{max}$, $\Delta\rho_{min}$ (e Å^{-3})	0.14, −0.16	0.24, −0.25	0.24, −0.22	0.20, −0.21
CCDC	2537564	2514808	2514809	2514810

Unit cell data at 293 K for phase II are *a* = 15.718(6) Å, *b* = 14.342(6) Å, *c* = 21.672(8) Å, α = 90°, β = 130.38(4)°, γ = 90°. Unit cell data at 293 K for phase III are *a* = 7.876(5) Å, *b* = 14.358(6) Å, *c* = 16.760(8) Å, α = 91.07(3)°, β = 96.38(4)°, γ = 99.56(5)°.

4.2 Morphological properties of crystals of phases I and II

Single crystals of phase I and phase II were mounted on the diffractometer and the orientation of the unit cell axes with respect to the macroscopic dimension of the crystals were determined. The faces of the crystals were also indexed. The results are shown in Fig. S6.

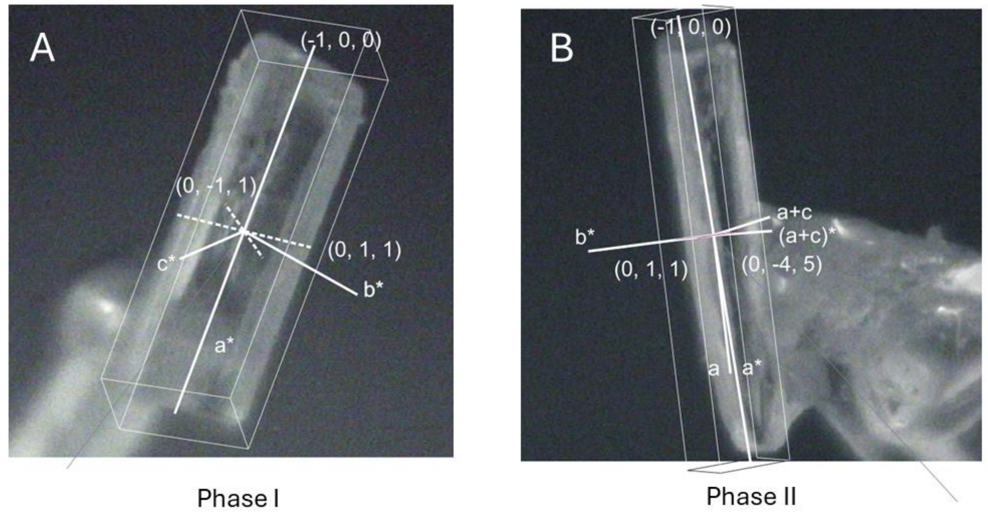

Fig. S6. Morphological properties of single crystals. A: phase I; B: phase II.

In both cases, single crystals have the habitus of elongated prisms. The direction of elongation of the prisms is always, for both phases, that of **a** axis of the unit cell. So, top and bottom faces are $(100)/(\bar{1}00)$. For phase I the lateral faces of the prisms are $(011)/(0\bar{1}\bar{1})$ and $(0\bar{1}1)/(01\bar{1})$. Other exposed lateral faces found in other crystallized samples are also (0 1 0)/(0 $\bar{1}$ 0) and (0 0 1)/(0 0 $\bar{1}$). The couple of lateral faces $(011)/(0\bar{1}\bar{1})$ is present also in the crystals of phase II.

4.3 Crystal structure of phase III

The crystal structure of form III, which is not immediately relevant to the dynamic properties of HMBB·NMP, was also determined, and is shown in Fig. S7. Phase III is triclinic, space group $P\bar{1}$, with lattice parameters (-100 °C) a = 7.786(3) Å, b = 14.321(3) Å, c = 16.686(3) Å, α = 91.048(16)°, β = 96.35(2)°, γ = 99.57(3)°, V = 1822.1(8) Å^3, Z = 4. Two imine molecules and two disordered NMP molecules are crystallographically independent. Metrically, the unit cell of phase III is similar to phase I, however, there is no relation between the two lattices, nor between the lattice of phase III and phase II. The pattern of H bonds in phase III is analogous to phases I and II, and the arrangement of NMP molecules is like phase II.

Heating crystals of phase III results in loss of NMP solvent molecules and formation of pure HMBB.

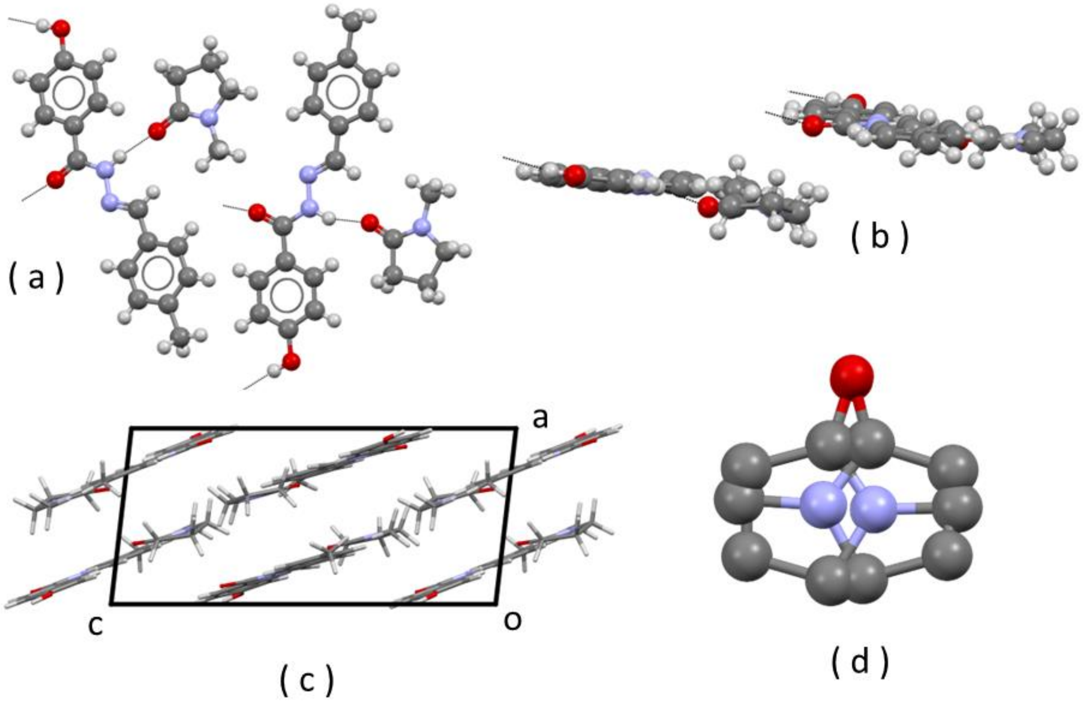

Fig. S7. Crystal structure of phase III. (a) face view of the independent unit (only one position of the disordered NMP molecules is shown); (b) independent unit viewed approximately along the line from the phenolic oxygen to the terminal C methyl; (c) packing of phase III viewed down **b**; (d) the two split positions of the disordered NMP molecules (H atoms excluded for clarity). The same type of disorder is present in phase II.

4.4 H-bonding in phases I, II and III

In Fig. S8 are shown the H-bonded chains/ribbons in the three polymorphs of HMBB · NMP.

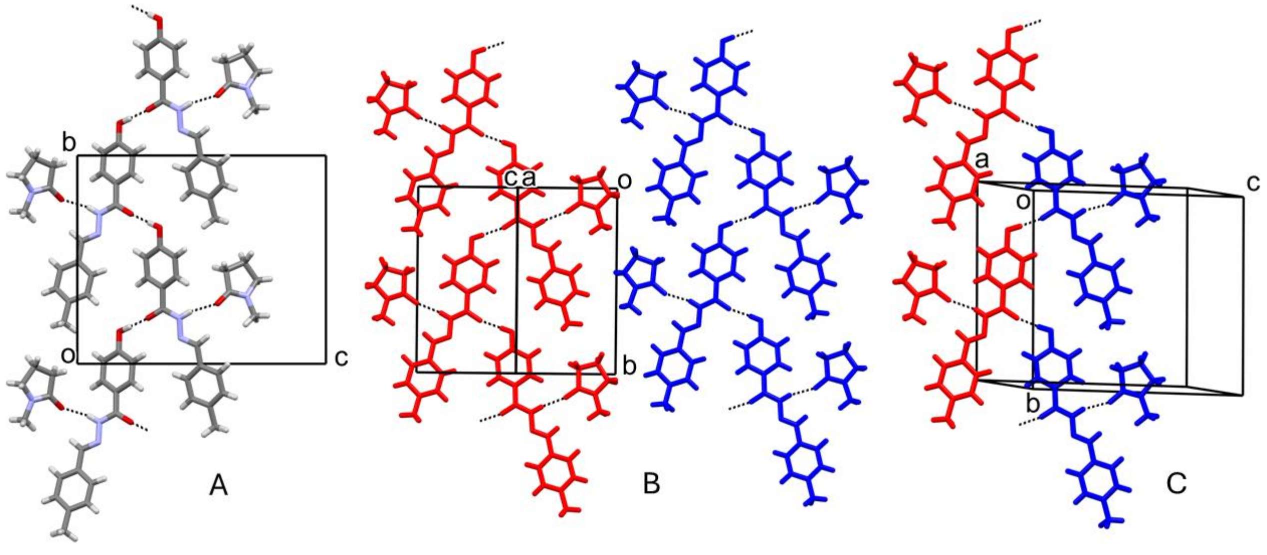

Fig. S8. H-bonds in the three polymorphs of HMBB · NMP. (A) H-bonded chain of phase I viewed down **a**; (B) two H-bonded chains of phase II viewed down **a**-**c**; (C) H-bonded chain of phase III. In (B) and (C) the two crystallographically independent molecules are shown in red and blue. Only one component of the disordered NMP molecules is shown in (B) and (C).

The topology of H-bonds is basically preserved in the three polymorphs. H-bonded chains are formed by H-bond between O-H donor of a molecule and carbonyl O acceptor the molecule following along the chain. The chains are laterally decorated with NMP molecules that are bonded to the chain through H-bonds between amide N-H donors of the chain and carbonyl O acceptor of the NMP molecules. This supramolecular architecture can be better described as a ribbon. In polymorphs I and II the ribbons are wrapped around 2_1 screw axes; in polymorph III they are generated by translation. As is shown in Fig. S8B, in polymorph II each independent molecule form chains with similar molecules (i. e. red with red and blue with blue). In phase III, instead, alternate red and blue molecules are present along each chain, as shown in Fig. S8C.

4.5 Metric and symmetry relations between phases I and II

The column of lattice vectors of the monoclinic supercell of phase I is obtained by applying the matrix M_I to the column of the orthorhombic lattice vectors

$$M_I = \begin{pmatrix} 4 & 0 & 1 \\ 0 & 1 & 0 \\ -8 & 0 & 1 \end{pmatrix}, detM_I = 12 \qquad (1)$$

In a similar way, the column of lattice vectors of the monoclinic supercell of phase II is obtained by applying the matrix M_{II} to the column of the monoclinic lattice vectors

$$M_I = \begin{pmatrix} 2 & 0 & 0 \\ 0 & 1 & 0 \\ 0 & 0 & 3 \end{pmatrix}, detM_I = 6 \qquad (2)$$

In both cases, the supercell contains Z=48 HMBB·NMP units.

The lattice parameters of the supercells (at -100 °C) are given in Table S2.

Table S2. Monoclinic supercells of phases I and II at -100 °C. Supercell parameters are in Å and °, volume in Å^3.

	a'	b'	c'	β'	Z'	V'
I	34.158	14.380	61.484	133.54	48	21891
II	30.768	14.307	63.885	129.01	48	21853

In Fig. S9A the crystallographic unit cells of phases I and II, along the common axis **b**, are shown, with indication of crystallographic symmetry elements. The orientation of the unit cell of phase I is consistent with the matrix transformation (1).

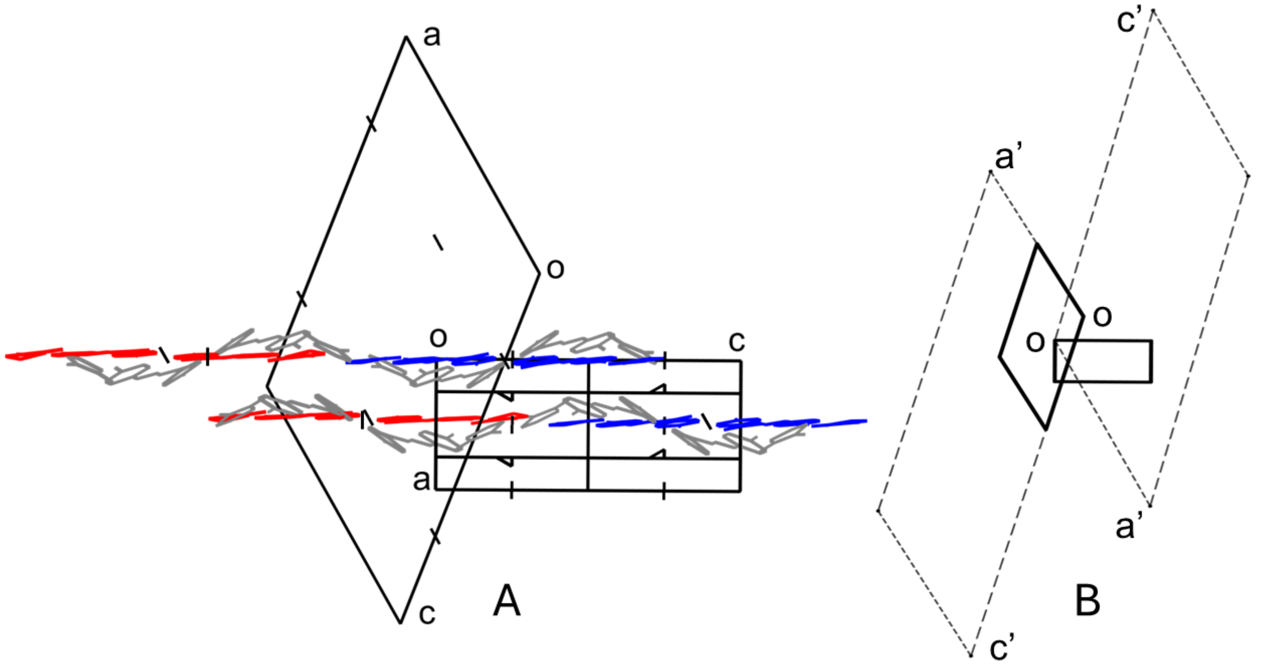

Fig. S9. A: View down the common axis **b** of superposition of phase I (molecules in gray) and phase II (molecules in red and blue), with indication of the crystallographic symmetry elements (screw axes only). H atoms omitted for clarity. Only one split position of the disordered NMP molecules is shown for phase II. B: Bare crystallographic unit cells (solid lines) and supercells (dashed lines) of phases I and II.

From the Fig. S9A it is evident that the 2_1 screw axes parallel to **b** of the orthorhombic lattice (phase I) involved in the formation of the H-bonded chains are kept in the monoclinic lattice (phase II). In particular 2_1 axes at (a=0 c=1/4) and (a=1/2, c=-1/4) of phase I are almost coincident with the 2_1 axis of phase II at (a=0 c=1/4) and (a=1/2 c=3/4). On the other hand, the 2_1 axes of phase I at (a=1/2 c=3/4) and (a=0 c=-3/4) are shifted, by about 2.6 Å along **c**, with respect to the corresponding axes of phase II at (a=-1 c=-1/4) and (a=1/2 c=1/4) respectively. In Fig. S9B bare crystallographic unit cells and supercells are shown. The metric similarity between phases I and II is evident.

Supercells are also very useful to rationalize the (small) elongation of crystals at the transition, as measured experimentally. Crystals of phase I are elongated in the **a** direction of the orthorhombic cell, and those of phase II in the **a** direction of the monoclinic cell (Fig. S6). In terms of supercells of Tab. S2, according to Fig. S9B, the two data to be compared are $a' cos30° = 29.581$ Å for phase I and $a' = 30.768$ Å for phase II. The calculated elongation is 4%.

4.6 Analysis of internal motions in phase I by X-ray diffraction data

It is well known that an accurate crystallographic analysis can be used to get information about overall and internal motion of molecules in crystals.[7,8] This information is encoded in the refined anisotropic displacement parameters (ADPs). Programs to retrieve this information are available for

the community of crystallographers. We have used the classic program THMA11 developed by Shomaker and Trueblood.[9] One important output of this program is the matrix of Δ values for couple of atoms, where

$$\Delta_{A,B} = \langle u_A^2 \rangle - \langle u_B^2 \rangle$$

$\Delta_{A,B}$ is the difference between the mean-square displacement amplitudes (MSDAs) of two atoms. For two atoms A and B belonging to a rigid group, $\Delta_{A,B}$ must be zero along the interatomic direction. So, from a close inspection of the matrix of Δ values, we can establish if the whole molecule behaves as a rigid group or if there are, in the molecule, subgroups of atoms that can be considered rigid. An ORTEP view of the independent unit of phase I at -100 °C is shown in Fig. S10.

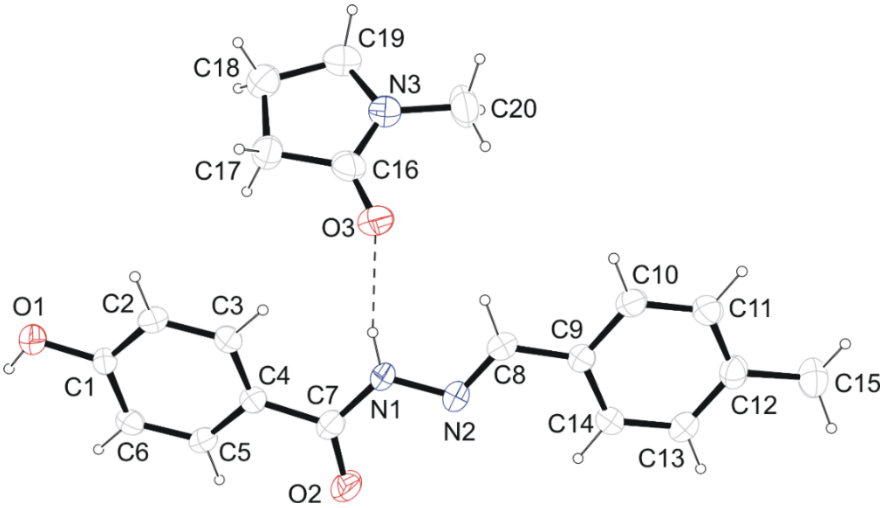

Fig. S10. ORTEP view of the independent unit of phase I at -100 °C. Anisotropic displacement parameters are reported at 50% probability level. H-bond is shown with dashed line.

The matrices of Δ values for the crystal structure of phase I at -100 °C and 20 °C are reported in Tables S3 and S4 respectively. For atomic numbering see Fig. S10. In Tables S3 and S4 we have collected under R1, R2 and R3 the entries related, respectively, to the phenyl ring C1 to C6, to the phenyl ring C9 to C14 and to the NMP molecule.

Table S3. Matrix of Δ values (pm^2) for phase I at -100 °C. Positive Δ means that MSDA along the interatomic vector $\overrightarrow{AB}$ is larger for B than for A; negative Δ means the reverse.

		R3								R2								R1								
	ATOM->	C20	C19	C18	C17	C16	N3	O3	C15	C14	C13	C12	C11	C10	C9	C8	C7	C6	C5	C4	C3	C2	C1	N2	N1	O2
	POSN->	1	1	1	1	1	1	1	1	1	1	1	1	1	1	1	1	1	1	1	1	1	1	1	1	1
	O1	53	167	186	112	104	105	196	48	48	46	38	89	94	19	67	18	45	-20	7	4	72	-44	-11	-41	20
	O2	231	24	109	137	24	26	35	-60	-87	-23	-62	-60	-24	-87	-8	42	30	-20	25	27	-15	-53	-28	-17	
	N1	343	103	163	175	110	103	48	79	98	87	71	88	98	44	94	60	37	53	18	78	96	-9	37		
	N2	379	103	129	129	115	80	25	39	58	52	32	27	32	-9	1	41	32	39	4	40	60	-38			
R1	C1	140	207	245	176	126	155	222	93	92	94	84	130	139	65	117	62	-28	-14	52	24	31				
R1	C2	61	134	218	135	59	87	142	-11	2	-15	-21	26	26	-42	-2	6	-19	19	44	-24					
R1	C3	197	120	187	169	104	137	207	31	25	12	23	85	94	5	51	-13	8	42	46						
R1	C4	227	127	187	169	56	114	118	38	36	46	32	70	91	16	80	8	22	33							
R1	C5	152	116	190	171	25	79	101	-8	-13	19	-17	5	30	-40	30	-6	49								
R1	C6	166	190	237	204	102	146	193	0	-11	10	-5	44	71	-15	60	-10									
	C7	227	55	128	130	10	39	3	-12	-9	15	-17	-6	15	-47	13										
	C8	503	167	136	109	187	147	84	-8	41	-18	-16	-3	3	-35											
R2	C9	464	185	124	96	182	130	104	28	26	-18	21	37	35												
R2	C10	427	196	95	46	161	123	86	14	-1	-7	23	13													
R2	C11	261	150	39	-3	104	50	59	0	-22	32	47														
R2	C12	269	179	86	50	144	77	103	4	-42	6															
R2	C13	376	204	128	96	179	126	128	-36	-6																
R2	C14	384	130	70	45	112	70	51	24																	
	C15	115	99	15	-3	92	-2	82																		
R3	O3	94	20	71	15	131	47																			
R3	N3	-95	-7	1	-25	-27																				
R3	C16	-47	-69	-82	-103																					
R3	C17	-72	10	28																						
R3	C18	-61	58																							
R3	C19	-27																								

T = -100 °C

Table S4. Matrix of Δ values (pm^2) for phase I at 20 °C. Positive Δ means that MSDA along the interatomic vector $\overrightarrow{AB}$ is larger for B than for A; negative Δ means the reverse.

		R3												R2								R1				
	ATOM->	O3	N3	C20	C19	C18	C17	C16	O2	O1	N2	N1	C15	C14	C13	C12	C11	C10	C9	C8	C7	C6	C5	C4	C3	C2
	POSN->	1	1	1	1	1	1	1	1	1	1	1	1	1	1	1	1	1	1	1	1	1	1	1	1	1
R1	C1	372	195	239	326	462	316	173	29	-1	-17	-10	82	122	73	17	71	160	25	89	13	-14	-23	16	-4	22
R1	C2	344	148	200	232	403	277	129	-7	-71	-60	-68	29	87	14	-26	30	105	-30	30	-6	-33	4	36	36	
R1	C3	363	158	345	150	303	289	129	-122	-42	-105	-116	14	46	-23	-41	38	127	-38	28	-94	22	50	-9		
R1	C4	256	164	424	193	349	346	110	-32	-19	-23	5	64	95	58	-8	35	139	11	82	-6	14	59			
R1	C5	225	94	297	157	328	313	55	22	16	-78	-45	4	15	11	-82	-56	52	-64	7	-39	29				
R1	C6	369	201	314	298	439	373	176	-2	-30	-43	-3	38	50	31	-37	19	132	-8	71	-15					
	C7	39	32	415	38	199	234	-10	-85	-15	-52	-43	28	56	39	-56	-49	48	-49	5						
	C8	109	243	834	241	216	175	207	73	-93	21	-71	-3	74	-33	-53	-40	46	-58							
R2	C9	193	283	818	331	246	184	246	162	-31	2	-19	55	44	-2	4	-7	33								
R2	C10	142	225	702	314	158	52	169	71	-167	-61	-117	-14	-21	-6	15	-45									
R2	C11	167	162	468	295	131	37	144	183	-85	9	-29	-24	-29	1	29										
R2	C12	199	200	491	336	191	104	185	191	-26	-7	-18	50	27	42											
R2	C13	199	253	650	345	231	157	214	83	-76	-81	-85	19	45												
R2	C14	59	164	682	221	127	66	103	131	-130	-141	-153	32													
	C15	172	98	270	244	105	29	117	88	-88	-83	-86														
	N1	67	153	600	139	260	290	121	-2	9	8															
	N2	19	121	641	124	197	211	106	80	12																
	O1	374	148	134	289	379	208	160	32																	
	O2	85	17	406	-2	188	267	22																		
R3	C16	-115	39	-13	-62	-44	-117																			
R3	C17	-15	27	-85	-15	22																				
R3	C18	-105	-12	-132	56																					
R3	C19	8	31	-60																						
R3	C20	-153	141																							
R3	N3	-22																								

T = 20 °C

From the two tables it is clear that the three rings can be considered rigid, because the correspondent Δ values (enclosed in triangles) are rather small, in particular for the two phenyl rings, and are both positive and negative. The three rectangular blocks contain instead Δ values for pair of atoms belonging to different rings. In the case of the block R1/R2 the values are relatively

small and both positive and negative, this indicating that for the two phenyl rings there is no relevant motion relative to one another. The two phenyl rings behave approximately as a whole rigid group. On the other hand, the two blocks R1/R3 and R2/R3 contain significantly higher values, all positive. This is clear evidence of significant motion of ring R3 relative to both R1 and R2. The motion increases, on heating from -100 °C to 20 °C, as evidenced by the entries that, in many cases, are almost doubled. While this internal motion of ring R3 with respect to the phenyl rings is expected, owing to the solvate nature of the crystal, nevertheless it can be prodrome of the local motions of the NMP that take place during the SCSC transition, as detailed in the typescript. We have also calculated the overall mean-square libration amplitude of the NMP molecule about the direction of the N1---O3 H-bond. It is $9.9(°)^2$ at -100 °C and $18.5(°)^2$ at 20 °C.

5. Raman measurements

5.1. Experimental details

Raman spectra of the biphasic frozen crystal (Figure 4A of the typescript) were acquired using a 633 nm laser source with a LabRAM HR Evolution spectrometer (HORIBA Scientific, France). The incident laser beam was focused on the sample by a 50xLWD objective (Olympus) with a numerical aperture of 0.50. Backscattered radiation was collected with a Synapse Plus BIDD Detector (1024 x 256 pixels), utilizing a 1800/nm grating. ULF-633 filter was applied to allow measurements up to 10 cm^{-1} of the spectrum. The laser power was set at 100%. Spectra were acquired with a 5 second acquisition time for 5 accumulations and a step-size of 0.8 μm. The scan was performed through a series of tangent circular focal spots running across the interface, as shown in Fig. 4B of the typescript and in Fig. S11.

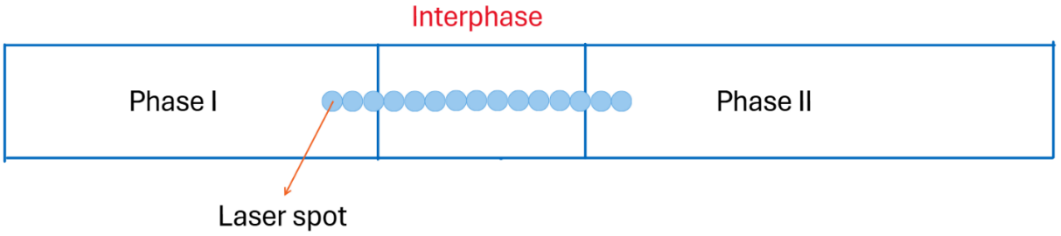

Fig. S11. Sketch of the space resolved Raman spectroscopy analysis of a frozen biphasic crystal of HMBB·NMP.

5.2. Discussion

The fact that, as shown in Fig. 4C of the typescript and Fig. S12, by running across the interface, the change of the Raman spectrum between that of pure phase II and pure phase I takes more than one step (nine for the record) is proof that the size of the interface is larger than the laser spot size.

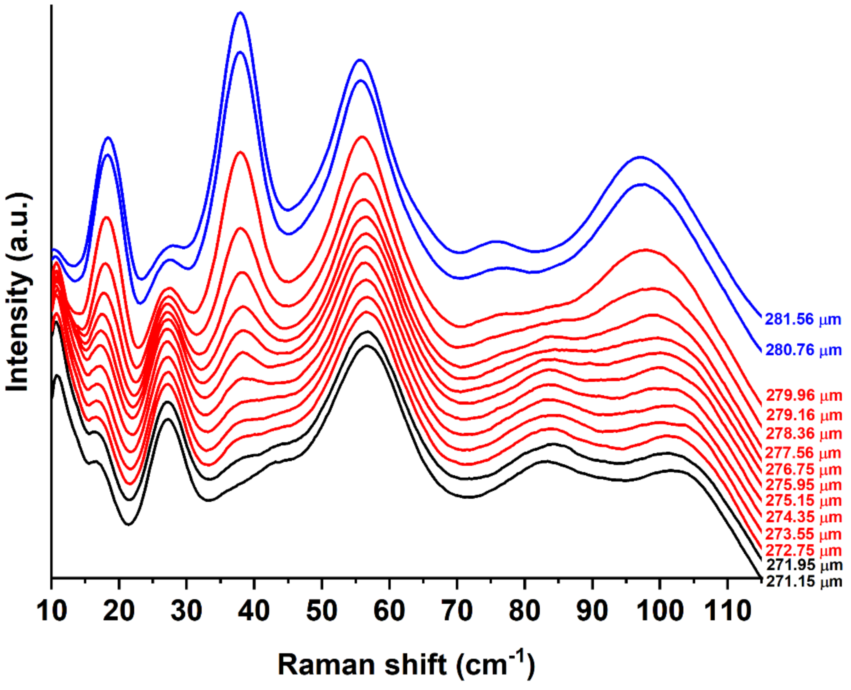

Fig. S12. Raman spectra recorded at 0.8 μm step across the interphase region.

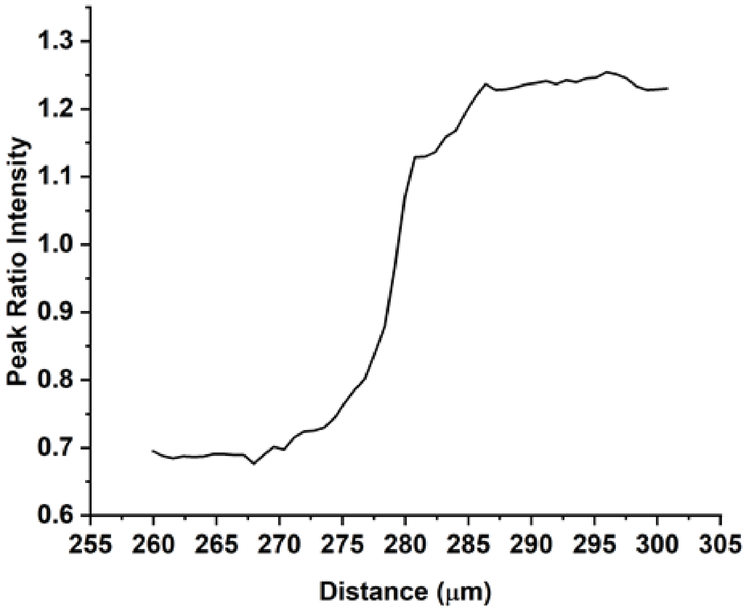

Fig. S13. Plot of the ratio of the intensity of the peak at 37.60 cm^{-1} to the peak at 55.77 cm^{-1} in every normalized spectrum, as a function of the distance along the crossing line of Fig. 4B of the typescript.

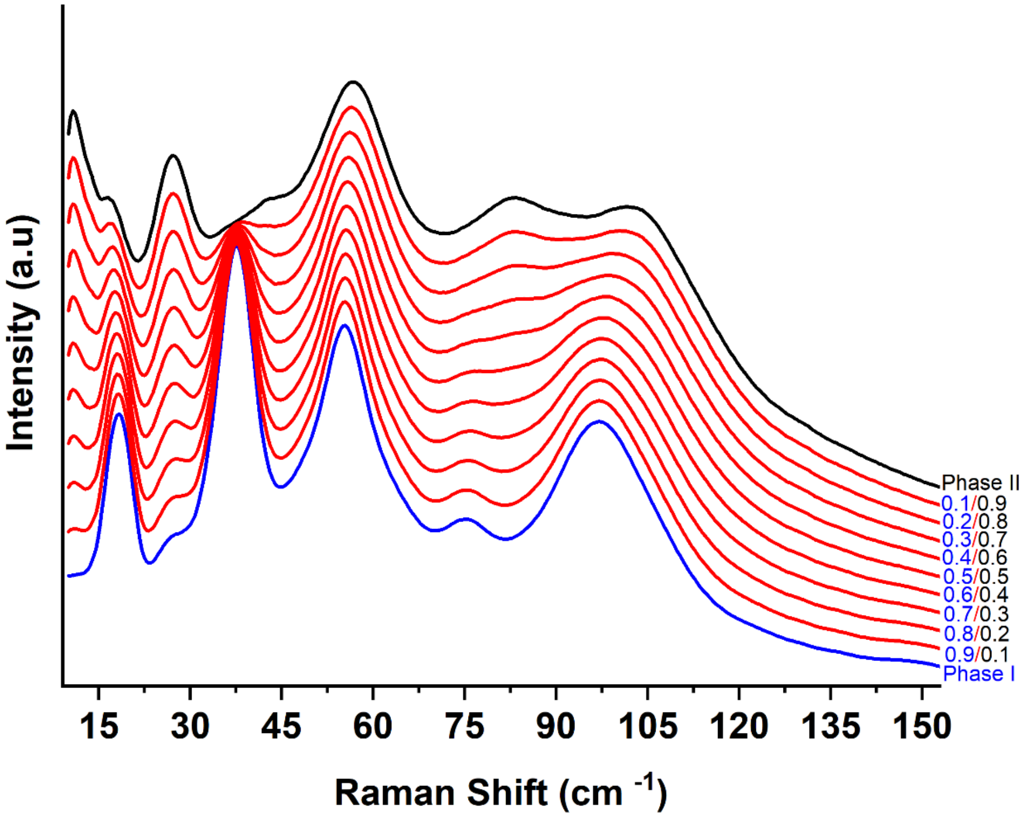

Fig. S14. Experimental Raman spectrum of phase I (blue) and phase II (black). The red spectra are calculated by summing the blue and black spectra, each multiplied by the proper molar fraction as indicated on the right end of the spectrum.

6. Nanoindentation measurements

Prior to nanoindentation measurements, a comprehensive crystallographic characterization of the HMBB·NMP single crystals was performed. The unit cell parameters and crystallographic orientations of the prominent crystal faces were determined using single-crystal X-ray diffraction analysis, followed by face indexing. This structural assessment provided an essential framework for correlating the measured nanoindentation responses with the crystallographic directions and anisotropic properties of the crystals. Nanoindentation measurements were performed using an Agilent G200 nanoindenter equipped with an XP head and a Berkovich diamond indenter. The experiments employed the continuous stiffness measurement (CSM) technique, allowing mechanical properties to be continuously evaluated as a function of indentation depth. Indentations were conducted to a selected depth at a constant strain rate of 0.05 s^{-1}, with a superimposed dynamic oscillation amplitude of 2 nm and a frequency of 45 Hz. The stiffness and area function of the indenter tip were calibrated using a fused silica reference sample (Corning 7980, Nanomechanics S1495-25). This calibration ensured accurate determination of hardness and elastic modulus during the nanoindentation measurements. Indentation experiments were carried out on selected crystallographic faces to a prescribed penetration depth of 500 nm. A Poisson's ratio of 0.30 was assumed for the material during data analysis. The mechanical properties, along with the projected contact area, were extracted from the corresponding load–displacement curves using the Oliver–Pharr analysis method.[10,11]

The residual indentation imprints on the HMBB·NMP crystals were characterized using atomic force microscopy (AFM) with a Bruker Dimension Icon instrument. Imaging was carried out in quantitative nanomechanical mapping (QNM) mode employing a ScanAsyst Air probe with a nominal spring constant of 400 mN m^{-1} and an estimated tip radius in the range of 2–12 nm. Data acquisition was performed using automated setpoint and feedback gain controls, with the software dynamically adjusting the parameters to minimize the trace–retrace error signal during scanning. Typical feedback conditions included force setpoints on the order of 30–40 nN and a feedback gain of approximately 6, although these values varied locally depending on surface conditions. Post-acquisition image processing, including mean plane subtraction for leveling and extraction of line profiles from the raw topography data, was conducted using Gwyddion software.[12]

7. Computational analysis

The CRYSTAL23[13] software package was utilized to perform solid-state density functional theory simulations on the three crystalline phases of HMBB·NMP starting from their experimental crystal structures. For crystal structures exhibiting disorder (phase II and phase III), the ideal ordered structure corresponding to the split position of NMP molecules with the highest occupancy was used. The PBE density functional[14] was employed along with the 6-311G(d,p) basis set.[15] This combination of density functional and basis set has been shown to be reliable for accurate simulations of molecular crystal structures and dynamics.[16,17] The PBE density functional was improved with D3 London-dispersion and Axilrod-Teller-Muto repulsion corrections in order to increase the accuracy of the intermolecular noncovalent interaction energies.[18-21] Due to the sensitivity and temperature-dependent nature of the polymorphs, the geometry optimizations were completed on only the atomic positions with the crystallographic lattice parameters fixed at 295 K experimental values. The calculations used a pruned DFT integration grid of 75 radial points and 974 angular points and a sampling of 64 k-points in the irreducible Brillouin zone. Energy convergence thresholds were set at $\Delta E < 10^{-8}$ Hartree for geometry optimizations and 10^{-10} Hartree for vibrational frequencies.

The relative electronic energies of the HMBB·NMP crystal phases were determined from the geometry optimizations. The energies in rank order from lowest to highest relative energy (per HMBB·NMP unit) are phase I = 0.0 kJ/mol, phase III = +2.2 kJ/mol, and phase II = +3.6 kJ/mol. Evidently, the metastable nature of phase I is related to a stabilization of phases II and III in terms of Gibbs free energy, because of the contribution of the configurational entropy due to the positional disorder of the NMP molecules.

Normal mode vibrational analyses were based on the numerical derivative of the Hessian matrix calculated with the central-difference formula using two displacements in Cartesian space of each atom in the crystallographic asymmetric unit cell. Computed Raman intensities were determined with the coupled-perturbed Hartree-Fock/Kohn-Sham (CPHF/KS) method,[22-24] with the final values adjusted for experimental laser wavelength (λ = 633 nm) and sample temperature (295 K).

Calculated frequencies of Phase I are included in the Table S5 below. The frequency analysis yielded no negative (imaginary) vibrational modes, indicating that the geometry optimized structure corresponds to a minimum on the potential energy surface. Animations of the three most prominent vibrations of Phase I in the sub-100 cm^{-1} region were produced using the Jmol program.[25] The lowest frequency mode at 20.19 cm^{-1} arises from a combined rotation around **b** of HMBB and NMP

molecules. The mode at 39.25 cm^{-1} is primarily an NMP rotational motion in the *bc*-plane. The third mode at 57.38 cm^{-1} is basically a bending vibration of HMBB and NMP molecules with respect to each other.

Table S5. Calculated vibrations for Phase I of HMBB·NMP up to 120 cm^{-1}. Raman intensities are normalized to a maximum value of 1000.0 (633 nm incident laser wavelength, temperature of 295 K).

Frequency (cm^{-1})	Mode Symmetry	Intensity (arb. units)
20.19	B1	221.48
24.05	A	22.40
27.66	A	97.26
28.98	B2	12.07
30.30	B3	15.64
32.13	B2	2.79
33.07	A	2.66
35.06	B1	6.33
37.62	B3	19.11
39.25	A	143.73
41.55	B3	0.15
42.94	B1	66.63
43.48	B2	5.97
46.74	B2	13.67
50.53	A	101.08
51.31	B1	22.06
53.20	B3	3.86
54.67	A	65.98
57.38	A	135.38
57.68	B2	12.21
58.32	B3	20.68
61.89	B1	0.02
63.15	B2	70.90
63.38	B1	3.96
64.67	A	33.68
66.20	B1	0.10
66.38	B3	23.31
67.43	B2	15.24
70.05	B3	0.12
73.88	A	55.18
76.46	B2	57.40
76.65	B3	53.70
77.54	B3	13.20
78.75	B2	22.58
79.39	A	12.55
79.81	B1	18.38
83.03	B2	13.78

83.79	B3	31.32
84.27	B1	4.90
84.97	B1	1.78
89.40	A	78.47
93.54	B2	1.59
95.32	A	17.49
97.41	B3	23.30
98.02	B1	22.56
101.71	B1	16.04
103.56	B2	9.49
104.03	B3	34.45
104.58	A	15.03
106.05	B2	2.52
107.05	A	130.14
111.55	B1	67.26
112.25	A	36.76
113.63	B3	2.25
113.96	B2	9.02
114.60	B3	19.53
116.27	A	15.76
117.89	B1	0.04

To facilitate comparison with the experimental Raman spectrum, the simulated spectrum has been convolved with a Lorentzian line shape (empirically determined) having a full-width-at-half-maximum (FWHM) of 6.0 cm^{-1}. The simulated and experimental spectra of phase I are shown in Fig. S15.

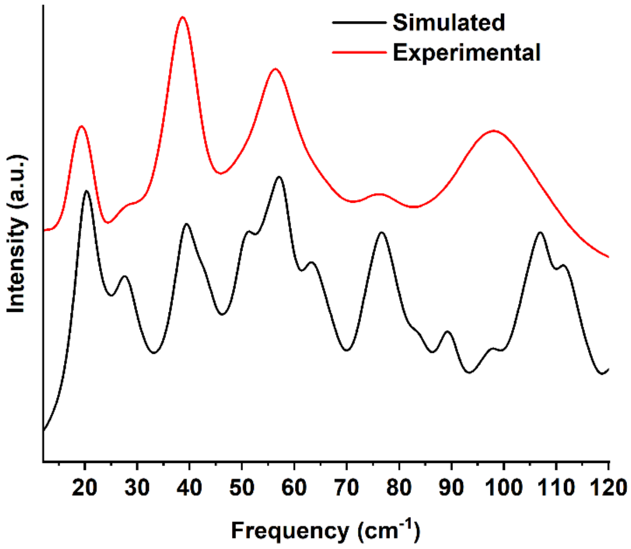

Fig. S15. Experimental and simulated Raman spectrum of phase I of HMBB·NMP.

8. References

(1) Parisi, E.; Borbone, F.; Carella, A.; Lettieri, S.; Capobianco, A.; Peluso, A.; Centore, R. Winning Strategy toward Acentric Crystals: Transverse Dipole Moment Molecules. *Cryst. Growth Des.* **2023**, *23*, 4538-4544.

(2) Bruker-Nonius (2002) SADABS, Bruker-Nonius, Delft, The Netherlands.

(3) Altomare, A.; Burla, M. C.; Camalli, M.; Cascarano, G. L.; Giacovazzo, C.; Guagliardi, A.; Moliterni, G. G.; Polidori, G.; Spagna R. SIR97: a new tool for crystal structure determination and refinement. *J. Appl. Crystallogr.* **1999**, *32*, 115-119.

(4) Sheldrick, G. M. Crystal structure refinement with SHELXL. *Acta Crystallogr.* **2015**, *C71*, 3-8.

(5) Farrugia, L. J. WinGX and Ortep for Windows: an update. *J. Appl. Crystallogr.* **2012**, *45*, 849-854.

(6) Macrae, C. F.; Bruno, I. J.; Chisholm, J. A.; Edgington, P. R.; McCabe, P.; Pidcock, E.; Rodriguez-Monge, L.; Taylor, R.; van de Streek, J.; Wood, P. A. Mercury CSD 2.0 – new features for the visualization and investigation of crystal structures. *J. Appl. Cryst.* **2008**, *41*, 466-470.

(7) Dunitz, J. D.; Maverick, E. F.; Trueblood, K. N. Atomic Motions in Molecular Crystals from Diffraction Measurements. *Angew. Chem. Int. Ed. Engl.* **1988**, *27*, 880-895.

(8) Dunitz, J. D.; Schomaker, V.; Trueblood, K. N. Interpretation of Atomic Displacement Parameters from Diffraction Studies of Crystals. *J. Phys. Chem.* **1988**, *92*, 856-867

(9) Schomacher, V.; Trueblood, K. N. On the Rigid-Body Motions of Molecules in Crystals. *Acta Crystallogr.* **1968**, B24, 63-76.

(10) Oliver, W. C.; Pharr, G. M. An Improved Technique for Determining Hardness and Elastic Modulus Using Load and Displacement Sensing Indentation Experiments. *J. Mater. Res.* **1992**, *7*, 1564-1583.

(11) Fischer-Cripps, A. C. Nanoindentation. Springer New York, NY, 2011.

(12) Nečas, D.; Klapetek, P. Gwyddion: an open-source software for SPM data analysis. *Cent. Eur. J. Phys.* **2012**, *10*, 181-188.

(13) Erba, A.; Desmarais, J. K.; Casassa, S.; Civalleri, B.; Donà, L.; Bush, I. J.; Searle, B.; Maschio, L.; Edith-Daga, L.; Cossard, A.; Ribaldone, C.; Ascrizzi, E.; Marana, N. L.; Flament, J.-P.; Kirtman, B. CRYSTAL23: A Program for Computational Solid State Physics and Chemistry. *J. Chem. Theory Comput.* **2023**, *19* (20), 6891-6932.

(14) Perdew, J. P.; Burke, K.; Ernzerhof, M. Generalized Gradient Approximation Made Simple. *Phys. Rev. Lett.* **1996**, *77* (18), 3865-3868.

(15) Frisch, M. J.; Pople, J. A.; Binkley, J. S. Self-consistent Molecular Orbital Methods 25. Supplementary Functions for Gaussian Basis Sets. *J. Chem. Phys.* **1984**, *80*(7), 3265-3269.

(16) Banks, P.; Song, Z.; Ruggiero, M. Assessing the Performance of Density Functional Theory Methods on the Prediction of Low-Frequency Vibrational Spectra. *J. Infrared Millim. Terahertz Waves* **2020**, *41*. https://doi.org/10.1007/s10762-020-00700-7.

(17) Parisi, E.; Santagata, E.; Kula, P.; Herman, J.; Gupta, S.; Simone, E.; Zarrella, S.; Korter, T. M.; Centore, R. Mechanical Transitions in Crystals: The Low-Temperature Thermosalient Transition of a Mesogenic Polyphenyl. *J. Am. Chem. Soc.* **2025**, *147* (17), 14731-14738.

(18) Grimme, S.; Antony, J.; Ehrlich, S.; Krieg, H. A Consistent and Accurate Ab Initio Parametrization of Density Functional Dispersion Correction (DFT-D) for the 94 Elements H-Pu. *J. Chem. Phys.* **2010**, *132* (15), 154104. https://doi.org/10.1063/1.3382344.

(19) Grimme, S.; Ehrlich, S.; Goerigk, L. Effect of the Damping Function in Dispersion Corrected Density Functional Theory. *J. Comput. Chem.* **2011**, *32*(7), 1456-1465.

(20) Grimme, S.; Hansen, A.; Brandenburg, J. G.; Bannwarth, C. Dispersion-Corrected Mean-Field Electronic Structure Methods. *Chem. Rev.* **2016**, *116*(9), 5105-5154.

(21) Axilrod, B. M.; Teller, E. Interaction of the van Der Waals Type Between Three Atoms. *J. Chem. Phys.* **1943**, *11*(6), 299-300.

(22) Ferrero, M.; Rérat, M.; Orlando, R.; Dovesi, R. Coupled Perturbed Hartree-Fock for Periodic Systems: The Role of Symmetry and Related Computational Aspects. *J. Chem. Phys.* **2008**, *128*(1), 014110. https://doi.org/10.1063/1.2817596.

(23) Ferrero, M.; Rérat, M.; Kirtman, B.; Dovesi, R. Calculation of First and Second Static Hyperpolarizabilities of One- to Three-Dimensional Periodic Compounds. Implementation in the CRYSTAL Code. *J. Chem. Phys.* **2008**, *129*(24), 244110. https://doi.org/10.1063/1.3043366.

(24) Ferrero, M.; Rérat, M.; Orlando, R.; Dovesi, R. The Calculation of Static Polarizabilities of 1-3D Periodic Compounds. the Implementation in the Crystal Code. *J. Comput. Chem.* **2008**, 29(9), 1450-1459.

(25) Jmol: an open-source Java viewer for chemical structures in 3D. http://www.jmol.org/
